# Halide Perovskites Breathe Too: The Iodide–Iodine Equilibrium and Self-Doping in Cs_2_SnI_6_

**DOI:** 10.1021/acscentsci.4c00056

**Published:** 2024-04-02

**Authors:** Julian A. Vigil, Nathan R. Wolf, Adam H. Slavney, Roc Matheu, Abraham Saldivar Valdes, Aaron Breidenbach, Young S. Lee, Hemamala I. Karunadasa

**Affiliations:** † Department of Chemistry, Stanford University, Stanford, California 94305, United States; ‡ Department of Chemical Engineering, Stanford University, Stanford, California 94305, United States; § Department of Physics, Stanford University, Stanford, California 94305, United States; ¶ Department of Applied Physics, Stanford University, Stanford, California 94305, United States; ∥ Stanford Institute for Materials and Energy Sciences, SLAC National Laboratory, Menlo Park, California 94025, United States

## Abstract

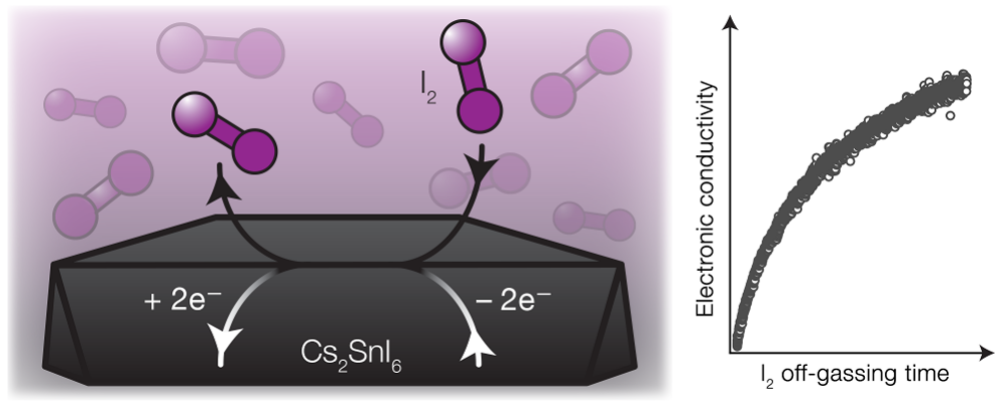

The response of an oxide crystal to the atmosphere can be personified as breathing—a dynamic equilibrium between O_2_ gas and O^2–^ anions in the solid. We characterize the analogous defect reaction in an iodide double-perovskite semiconductor, Cs_2_SnI_6_. Here, I_2_ gas is released from the crystal at room temperature, forming iodine vacancies. The iodine vacancy defect is a shallow electron donor and is therefore ionized at room temperature; thus, the loss of I_2_ is accompanied by spontaneous *n*-type self-doping. Conversely, at high I_2_ pressures, I_2_ gas is resorbed by the perovskite, consuming excess electrons as I_2_ is converted to 2I^–^. Halide mobility and irreversible halide loss or exchange reactions have been studied extensively in halide perovskites. However, the reversible exchange equilibrium between iodide and iodine [2I^–^_(*s*)_ ↔ I_2(*g*)_ + 2e^–^] described here has often been overlooked in prior studies, though it is likely general to halide perovskites and operative near room temperature, even in the dark. An analysis of the 2I^–^_(*s*)_/I_2(*g*)_ equilibrium thermodynamics and related transport kinetics in single crystals of Cs_2_SnI_6_ therefore provides insight toward achieving stable composition and electronic properties in the large family of iodide perovskite semiconductors.

## Abstract 2

We study the reversible exchange between I_2_ gas and iodide ions in a single crystal of the double perovskite Cs_2_SnI_6_. Measurements of bulk ion diffusion, electron transport properties, and thermodynamics of the 2I^−^_(*s*)_/I_2(*g*)_ equilibrium indicate that I_2_ off-gassing is spontaneous near room temperature and dopes the perovskite with excess electrons. Mitigating this reaction is critical to achieving stable electronic properties from perovskite semiconductors.

## Introduction

Compositional heterogeneity,^1−3^ mixed ionic–electronic conductivity,^4,5^ and photoinduced instabilities^6,7^ still present obstacles in realizing stable properties from halide perovskite (*ABX*_3_; *X* = halide) photovoltaics and optoelectronic devices. Efficiencies of perovskite solar cells have risen to an impressive 26.1% for single-junction cells and 33.9% for monolithic perovskite-Si tandem cells,^8^ yet champion devices do not maintain their efficiency over module-scale areas nor do they exhibit the low degradation rates of more established solar absorbers. Some lag in scalability and stability for perovskite-based devices is expected relative to more-established technologies;^9^ however, the primary origin of instabilities in perovskites appears to be intrinsic to the material composition and structure. Thus, a closer look at the thermodynamic stability and defect chemistry of halide perovskites is required to learn how to counteract reactions that degrade the material.

Halide perovskites—both as rapidly crystallized thin films and as slowly grown single crystals—exhibit significant crystallographic disorder including point defects (zero-dimensional imperfections in the lattice such as vacancies, interstitials, and substitutions). Large point defect concentrations, attributed to their small formation energies (in some cases less than 0.1 eV per defect pair;^10^*k*_*B*_*T* = 26 meV at 300 K), contribute to bulk ionic conductivity. Experiments demonstrate that the halogen vacancy is the dominant mobile defect in various perovskites, with vacancy migration activation barriers of less than 0.3 eV.^11^ The mobile halides produce ionic polarization effects in an external field, contributing to undesirable current–voltage hysteresis and other instabilities in devices.^4,5,12,13^ Furthermore, the ionic conductivity of iodide perovskites is enhanced under illumination,^14,15^ exacerbating adverse effects during device operation. Efforts to characterize halogen point defects are consequently paramount to controlling perovskite defect chemistry, particularly to stabilize ionic and electronic transport. To date, however, these studies are primarily limited to internal defect reactions dictated by crystallization conditions.

Oxides, including perovskites (*AB*O_3_), exhibit a rather unique external defect reaction: oxygen exchange. In defect notation, oxygen exchange describes an equilibrium between the pristine crystal (*nil*) and dicationic oxygen vacancies (*V*_*O*_^••^), gaseous oxygen, and electrons (*e*′):
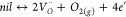
1

Equation 1 represents a case in which the oxygen vacancy is ionized and electrons are delocalized. In practice, the fate of the electron depends on the composition and state (external temperature/pressure) of the crystal. Electrons can be delocalized in the conduction band, for instance, in SnO_2_,^16^ or localized and ascribed to the formal reduction of a coordinated metal, as in La_2_CuO_4_^16,17^ and CeO_2_.^18,19^ The 2O^2–^_(*s*)_/O_2(*g*)_ equilibrium is central in characterizing oxide defect chemistry^20^ and manipulating oxygen nonstoichiometry, including for application as solid oxide conductors^21^ and high-*T*_*c*_ superconductors.^17^

All solids have nonzero (though typically very low) vapor pressure at ambient conditions, and many decompose to produce gaseous components at elevated temperatures. Alternatively, oxides constitute a large fraction of solids that could exhibit a reversible exchange defect reaction analogous to eq 1, without decomposition or phase change, where vacancy creation is coupled to the formation of a homonuclear diatomic molecule. At high temperatures (>500 K), oxides exhibit 2O^2–^_(*s*)_/O_2__(*g*)_ exchange and H_2_ gas can incorporate into metals and form hydrides [2H^–^_(*s*)_/H_2__(*g*)_], though the latter are often not single-phase transformations.^22^ Nonstoichiometry has been observed in nitrides,^23^ but 2N^3–^_(*s*)_/N_2__(*g*)_ exchange is unlikely to occur at modest temperatures due to the N≡N bond strength. This leaves only the halogens (*X*_2_) as homonuclear diatomic molecules that can reversibly react with ionic crystals at modest temperatures. Halogen bond enthalpies are significantly lower than those of O_2_, H_2_, and N_2_,^24^ which should facilitate *X*_2_ bond breaking at modest temperatures. Furthermore, metal–halogen bond enthalpies are lower than corresponding metal–oxygen bond enthalpies (except in the case of fluorides) and tend to be similar across *B*-site metals common to the main group perovskites, such as Pb, Sn, and Ge.^25^

Here, we present the first characterization of the iodide–iodine exchange equilibrium at the solid perovskite–gas interface through variable-temperature and variable-I_2_-pressure electronic property measurements of single crystals. This equilibrium dictates the concentration of conduction electrons, which profoundly affects the electronic properties of the perovskite. Given the importance of iodide perovskites, which feature suitable band gaps for photovoltaics, we sought to focus on their defect chemistry. We chose the double perovskite Cs_2_SnI_6_ as a case study due to its relatively simple defect chemistry—eliminating confounding defect reactions involving H^+^ and volatile organoamines found in the hybrid perovskites—and our ability to form large single crystals with well-defined terminations for bulk transport analyses. We apply a diffusion model that describes the transport of iodine vacancies, which facilitates iodine exchange between the bulk crystal and the atmosphere. Electronic property measurements show that the iodine vacancy is a shallow electron donor, and the material therefore self-dopes upon I_2_ loss. Further analysis of the thermodynamics of the 2I^–^_(*s*)_/I_2(*g*)_ equilibrium suggests the generality of this reaction to other iodide perovskites. We posit that halogen off-gassing and exchange are underappreciated defect reactions in this large class of technologically important materials, and controlling this reaction is imperative for obtaining stable optoelectronic properties from devices using these semiconductors.

## Prior Work

Reversible Br_2_ loss was reported by us to occur in an elpasolite-type double perovskite: Cs_2_AgTlBr_6_.^26^ The electronic conductivity of Cs_2_AgTlBr_6_ increased over time under flowing N_2_ near room temperature. In this case, *n*-doping was inferred, but not directly measured, from the loss of Br_2_ in the forward reaction (as in eq 1).

This reversible reaction does not require light, unlike the well-known photodecomposition of metal halides under irradiation, which formed the basis of black-and-white photography.^27^ Similar light-induced decomposition occurs in halide perovskite crystals (e.g., Cs_2_AgBiBr_6_^28^) and thin films [e.g., (CH_3_NH_3_)PbI_3_^29^], particularly under UV excitation. Further, halide exchange (e.g., *AB*I_3_ to *AB*Br_3_) has been explored extensively in perovskites and follows two predominant mechanisms: (*i*) exchange driven by redox chemistry in which the halide in a perovskite (e.g., I^–^) reacts irreversibly with a more oxidizing halogen gas (e.g., Br_2_)^30,31^ and (*ii*) where an excess halide source (typically as *X*^–^ or H*X*) can affect the exchange through mass action.^32,33^ These halide-exchange reactions, which require additional reactants and do not electronically dope the perovskite, are distinct from the spontaneous self-doping halogen loss we describe here.

Reversible I_2_ loss is also distinct from well-studied defect reactions involving iodides (I^–^), including internal defect pairs that form during crystallization (e.g., I^–^ vacancy and a CH_3_NH_3_^+^ vacancy),^4,34,35^ light-induced I_2_ loss via perovskite–solvent^14,36,37^ or −solid^14,38^ interfaces, and irreversible decomposition at electrodes.^39^ For example, iodine loss has been detected from (CH_3_NH_3_)PbI_3_ films immersed in toluene, which was greatly accelerated by light exposure.^14^ However, reactions at the perovskite–liquid interface are often complicated by even slight dissolution of the perovskite and by the fact that dissolved iodide is readily oxidized by light and O_2_. Maier and co-workers observed an increase in electronic conductivity upon I_2_ exposure of thin films of *p*-type (CH_3_NH_3_)PbI_3_,^34^ and Cahen and co-workers observed an increase in the semiconductor work function when *p*-type (CH_3_NH_3_)PbI_3_ thin films were exposed to I_2_ gas.^40^ These observations are consistent with a reduction in electron concentration (*p*-type doping) as I_2_ dissociates and incorporates into the perovskite as I^–^. To our knowledge, these observations constitute the closest precedent to the work described here, although the composition (particularly methylammonium) and abundant grain boundaries in polycrystalline (CH_3_NH_3_)PbI_3_ films introduce alternative reactivity and transport pathways for iodine gas. Thus, the chemistry of the 2I^–^_(*s*)_/I_2(*g*)_ equilibrium has not been probed and the reversibility, bulk thermodynamics, and transport consequences of the halide–halogen exchange equilibrium in halide perovskites have not been established in prior studies.

## Results

### Structure and Transport Properties of Cs_2_SnI_6_

**Figure 1:**
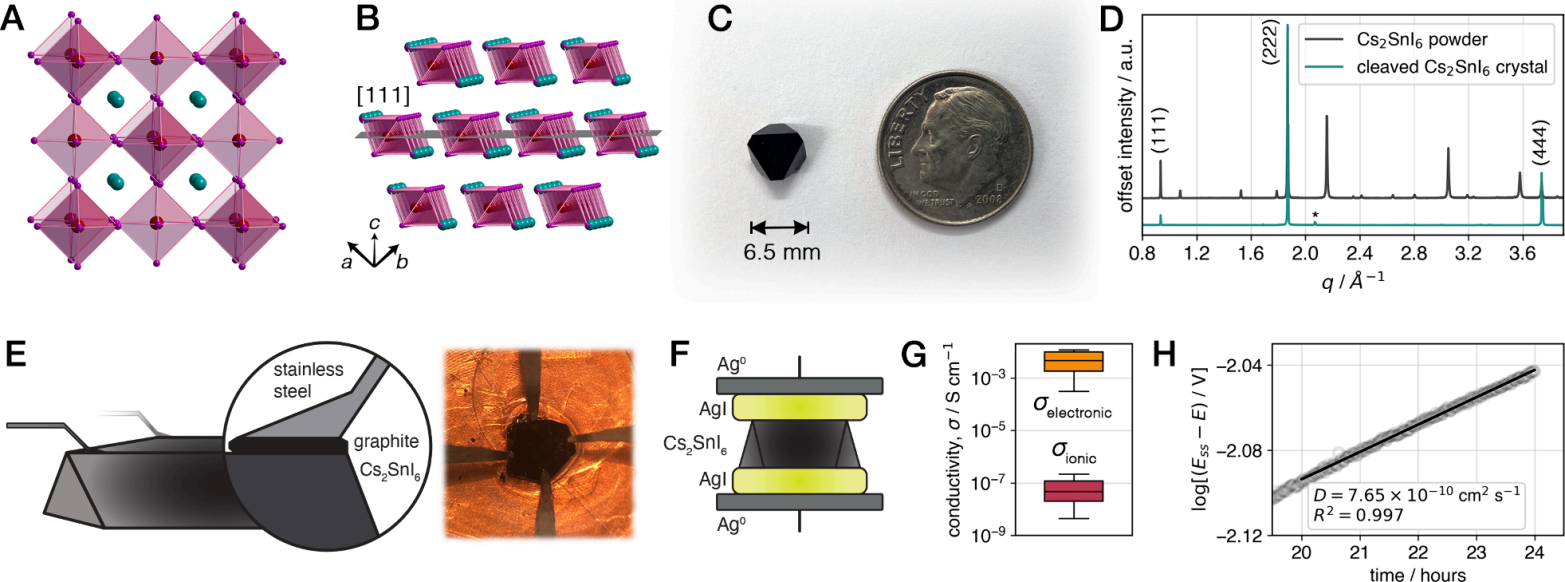
Growth and transport measurements of millimeter-scale Cs_2_SnI_6_ single crystals. Single-crystal X-ray structure of Cs_2_SnI_6_^41^ (*Fm–3m*) viewed along a principal axis (A) and parallel to the [111] plane, illustrating alternating tin iodide and vacancy layers (B). Tin-iodide octahedra are shaded in magenta, and Cs^+^ ions are shown in teal. (C) Photograph of a representative Cs_2_SnI_6_ crystal with {111} termination. (D) X-ray diffraction patterns of Cs_2_SnI_6_ crystals, either ground to a powder or cleaved; the {111} face-centered cubic (*Fm–3m*) Bragg peaks are labeled with the corresponding Miller indices, and * denotes a background peak from the crystal holder. (E) Schematic representation (left) and photograph (right) of a cleaved Cs_2_SnI_6_ crystal contacted in four-point geometry for an electronic conductivity measurement. (F) Schematic representation of a Cs_2_SnI_6_ crystal contacted in two-point geometry for an ionic conductivity measurement. (G) Box plots of the distribution of ionic and electronic conductivity measurements (*σ*_*ionic*_ and *σ*_*electronic*_) at room temperature; the box centerline represents the median, box limits represent upper and lower quartiles, and bar limits represent maximum and minimum values. (H) Relaxation behavior (open-circuit potential) following a symmetric direct-current polarization in the long-time limit; the bulk diffusion coefficient (*D*) is determined from a linear fit of the power law dependence (*E*_*ss*_ is the steady-state potential from the prior polarization).

The K_2_PtCl_6_-type double perovskite Cs_2_SnI_6_ crystallizes in a face-centered cubic (*Fm–3m*) structure^41^ (Figure 1A) with Sn^4+^ and vacancies alternating in the octahedral sites; the vacancy ordering is particularly evident when the structure is viewed parallel to a {111} plane (Figure 1B). Despite the lack of connectivity between tin iodide octahedra, Cs_2_SnI_6_ is a low-band-gap *n*-type semiconductor that exhibits electronic conductivity up to 10^–2^ S cm^–1^ and dispersive electronic bands,^42−45^ particularly in the low-lying conduction band with Sn 5*s* and I 5*p* character.^44^ The electronic properties of Cs_2_SnI_6_ therefore resemble those of the 3D iodide perovskites.

We found that millimeter-scale crystals of Cs_2_SnI_6_ could be grown from a saturated solution of the precursors in dry γ-butyrolactone upon slow cooling from 130 °C (Figure 1C; see Methods for details). Crystals terminate primarily along the {111} planes, yielding a truncated octahedral habit. Isolated crystals also cleave easily along {111} planes (Figure 1D), allowing for the preparation of thin plate-like crystals for ionic or electronic conductivity measurements. To our knowledge, this is the first reported method to form millimeter-scale single crystals of Cs_2_SnI_6_, ideal for bulk transport measurements without grain-boundary effects.

Single-crystal van der Pauw measurements (Figure 1E; see Methods) indicate an average electronic conductivity of 4.5 × 10^–3^ S cm^–1^ (Figure 1G and Figure S1, Supporting Information), consistent with measurements on polycrystalline pellets or thin films.^42−45^ All electronic property measurements used graphite contacts to eliminate undesired reactions at the surface of the crystal, which are often seen with metal electrodes. To quantify the ionic transport properties, we fabricated symmetric cells with electron-blocking, ion-conducting contacts (Figure 1F; see Methods for details). The potential drop across the cell corresponds to the ionic resistivity of Cs_2_SnI_6_, yielding an average ionic conductivity of 7.1 × 10^–8^ S cm^–1^ (Figure 1G and Figure S2, Supporting Information). The ionic conductivity and electronic conductivity differ by more than 10^4^; thus, Cs_2_SnI_6_ is not a mixed ionic–electronic conductor (Figure S3, Supporting Information).

The symmetric ion-conducting cell further allows for an estimation of the bulk ionic diffusion coefficient when the applied current is removed (see Methods).^46^ A linear fit of the power-law linearization of the measured open-circuit potential relaxation profile (see Supporting Methods and Figure S4, Supporting Information) yields a diffusion coefficient (*D*) of 7.65 × 10^–10^ cm^2^ s^–1^ (Figure 1H). A solid-state coulometry measurement^31^ (Figure S5, Supporting Information) further confirmed that the iodide anion (I^–^) is the dominant mobile ion in Cs_2_SnI_6_, in line with prior analyses of various 3D perovskites.^4,11^

### Electronic Equilibrium Accompanying 2I^–^_(*s*)_/I_2(*g*)_ Exchange

**Figure 2:**
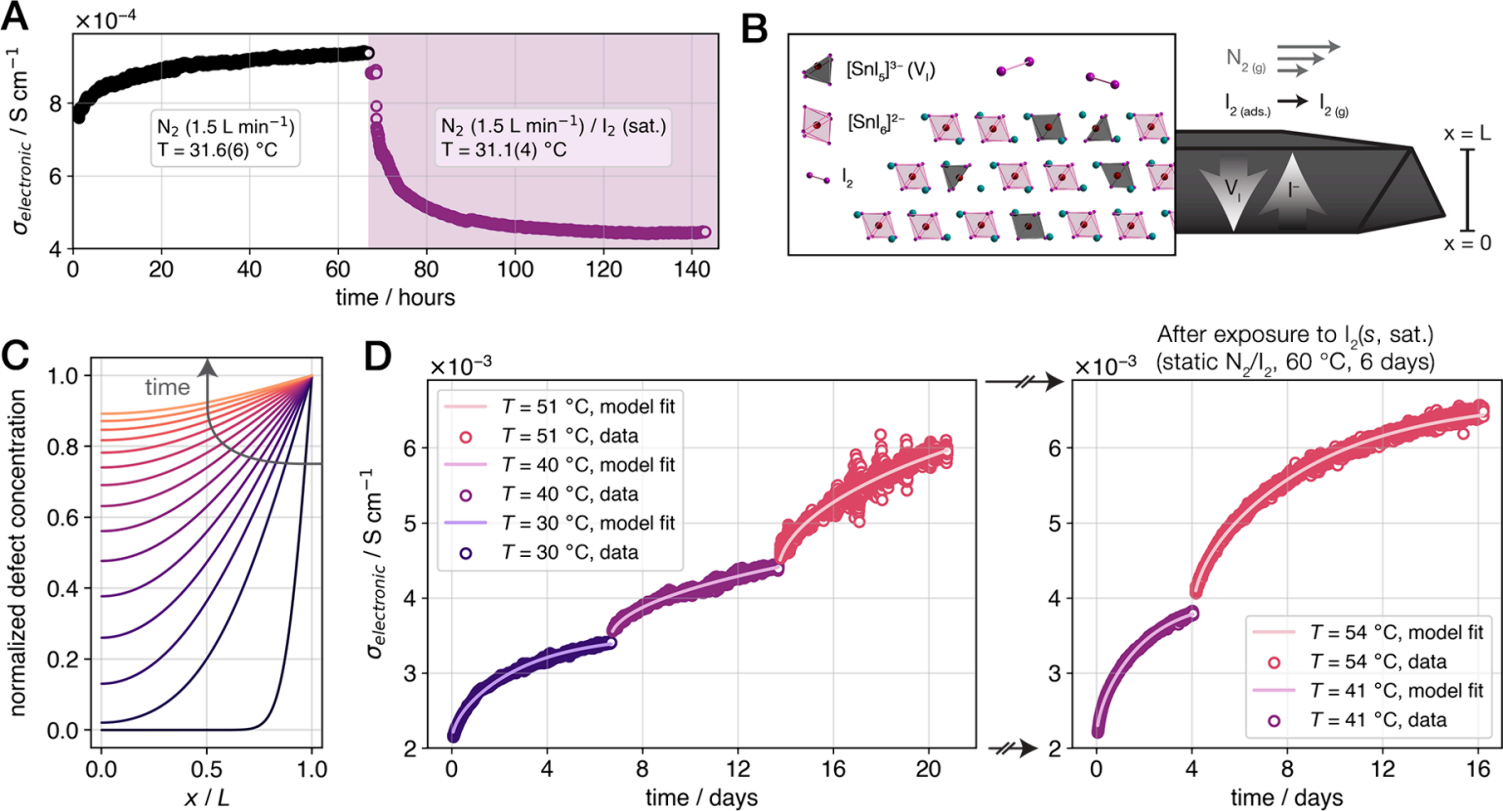
The 2I^–^_(*s*)_/I_2(*g*)_ equilibrium and modeling of the diffusion-limited kinetics. (A) Isothermal electronic conductivity measurements under flowing N_2_ (1.5 L min^–1^) for approximately 67 h, followed by the introduction of a column of solid I_2_ for the remainder of the measurement; the saturated vapor pressure of I_2(*s*)_ at 25 °C is 40.6 Pa.^47^ (B) Schematic representation of a cleaved Cs_2_SnI_6_ crystal with a dimensionless thickness of *L* and arrows indicating the direction of diffusion in the off-gassing reaction (forward reaction, eq 2) relative to the exposed surface of the crystal at *x* = *L*; a schematic representation of the local equilibrium at the crystal surface is given in the inset, where iodine vacancies are depicted without a change in the local structure of the resulting five-coordinate Sn centers. (C) Defect concentration profiles simulated from the solution to the one-dimensional diffusion model, where the *x* axis corresponds to the distance from the crystal base (*x* = 0) to the exposed surface (*x* = *L*); individual traces correspond to distinct values of dimensionless time, and the arrow indicates the progression from *t* = 0 to *t* → ∞. (D) Temperature-step off-gassing conductivity measurements and model fits (main text, eq 3) under flowing N_2_ (3 L min^–1^) for a pristine crystal (left panel), which was then transferred to a closed container with I_2(*s*, sat.)_ and held at 60 °C for 6 days; the crystal was removed, cleaved, and measured again under flowing N_2_ (right panel).

We regularly observed a monotonic increase in the electronic conductivity of Cs_2_SnI_6_ crystals, over hours to days, during continuous measurements under a flow of inert gas (e.g., Figure 2A, left). This response is characteristic of off-gassing,^26^ where the inert gas flow removes the buildup of a gaseous product on or near the crystal surface, further driving the reaction. Furthermore, as a representative Cs_2_SnI_6_ crystal approached steady-state electronic conductivity under flowing N_2_ at 40 °C (after 60 h; Figure 2A, left), we introduced a column of solid I_2_ to saturate the atmosphere with I_2_ vapor and tested the reversibility of the equilibrium. The conductivity decreases rapidly upon introduction of I_2_ and reaches a steady state within tens of hours (Figure 2A, right), notably equilibrating with a similar time constant as for the off-gassing response (Figure S6, Supporting Information).

Thus, we propose the following exchange defect reaction, by analogy to the oxides^16^ (eq 1),
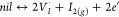
2where *V*_*I*_^•^ is the monocationic iodine vacancy. An alternative representation of the exchange equilibrium involving iodine interstitial defects has been contemplated,^40^ though the likelihood of interstitial contributions is significantly greater in (CH_3_NH_3_)PbI_3_ (than in Cs_2_SnI_6_) based on formation-energy calculations.^48,49^ The present work is consistent with the vacancy representation expressed in eq 2; alternative hypotheses can be ruled out on the basis of defect thermodynamics calculations and numerous indirect observations (detailed in Supporting Discussion S.D.1, Supporting Information). The external nature of the exchange defect equilibrium is represented schematically in Figure 2B, emphasizing the dual implications on the local defect structure and bulk transport when iodine vacancies and I_2_ are produced (or annihilated) at the surface of Cs_2_SnI_6_.

### Model for Diffusion-Limited 2I^–^_(*s*)_/I_2(*g*)_ Exchange Kinetics

An inspection of the conductivity profiles in Figure 2A and Figure S6 (Supporting Information) reveals a common time constant in the approach to steady state in both directions of the 2I^–^_(*s*)_/I_2(*g*)_ equilibrium (eq 2). This behavior is characteristic of diffusion-limited kinetics on the length scale of the bulk crystal. Thus, we applied a one-dimensional diffusion model to the system based on the flat-plate approximation.^50,51^ Here, the Cs_2_SnI_6_ crystals are cleaved to thin plates with in-plane dimensions that are significantly larger than the thickness (*x* = *L*, Figure 2B). A detailed treatment of the diffusion model is given in the Methods section; briefly, an expression for the bulk electronic conductivity (σ) takes the following form,
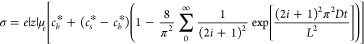
3where *e* is the elementary charge, *z* is the charge of the defect, *μ*_*e*_ is the electron mobility, *c*_s_* and *c*_b_* are the excess defect concentrations at the surface and in the bulk, respectively, *t* is time, and *L* is the thickness of the crystal. The defects described by the model are ionized iodine vacancy defects produced in the external off-gassing reaction (see Self-Doping: Iodine Vacancies as Shallow Electron Donors); concentration values therefore represent additional vacancies beyond the internal vacancy concentration—set during the crystallization and before exposure to the open atmosphere (see Supporting Discussion S.D.2)—and we therefore refer to these quantities as excess concentrations.

The time-dependent response, captured in eq 3, follows from a change in the environment that produces a response—first at the surface of the crystal—that drives bulk diffusion (Figure 2B). For instance, an increase in temperature or a decrease in the external partial pressure of I_2_ [*p*(I_2_)] shifts the equilibrium toward off-gassing (eq 2, forward reaction). The surface equilibrates instantaneously with the atmosphere and sets the maximum iodine vacancy concentration. As time passes, these vacancies diffuse into the bulk of the crystal and I^–^ ions diffuse toward the surface, where the surface again equilibrates with the atmosphere. This process continues until the concentration profile levels (Figure 2C) and the crystal reaches a steady state. The magnitude of the concentration gradient is proportional to the rate of change of the bulk electronic conductivity; thus, the conductivity is expected to plateau as time tends to infinity.

We refined the model (eq 3) against the experimental off-gassing conductivity traces at multiple temperatures (Figure 2D) by varying *D*, *c*_s_*, and *c*_b_* (see Methods and Supporting Information). The refinement yields excellent agreement between model fits and the experimental data without strict limits or initial guesses, exemplified by both panels of Figure 2D. The optimized values of *D*, *c*_s_*, and *c*_b_* for these fits are given in Table S1. Notably, the fit values of *D* are in the range of 2 × 10^–10^ cm^2^ s^–1^ to 1 × 10^–9^ cm^2^ s^–1^, in agreement with quantification from solid-state polarization measurements (Figure 1H).

To confirm the reversibility of the equilibrium, as demonstrated by the in-line flow experiment (Figure 2A), we subjected the measured Cs_2_SnI_6_ crystal (Figure 2D, left) to a high-*p*(I_2_) treatment (see Methods) before reproducing the original measurement conditions. The electronic conductivity returns to near the original state after treatment (Figure 2D, right), reflecting the shift in the equilibrium to favor the reverse reaction (eq 2) upon exposure to saturated *p*(I_2_). The conductivity traces then mirror the original measurement, as the system again tends toward the off-gassed state under flowing N_2_. The time constant of the conductivity response after the high-*p*(I_2_) treatment—and subsequently cleaving the crystal while maintaining the exposed surface area—changes in accordance with the reduced crystal thickness, another hallmark of diffusion-limited kinetics (Figure S7, Supporting Information).

### Self-Doping: Iodine Vacancies as Shallow Electron Donors

**Figure 3:**
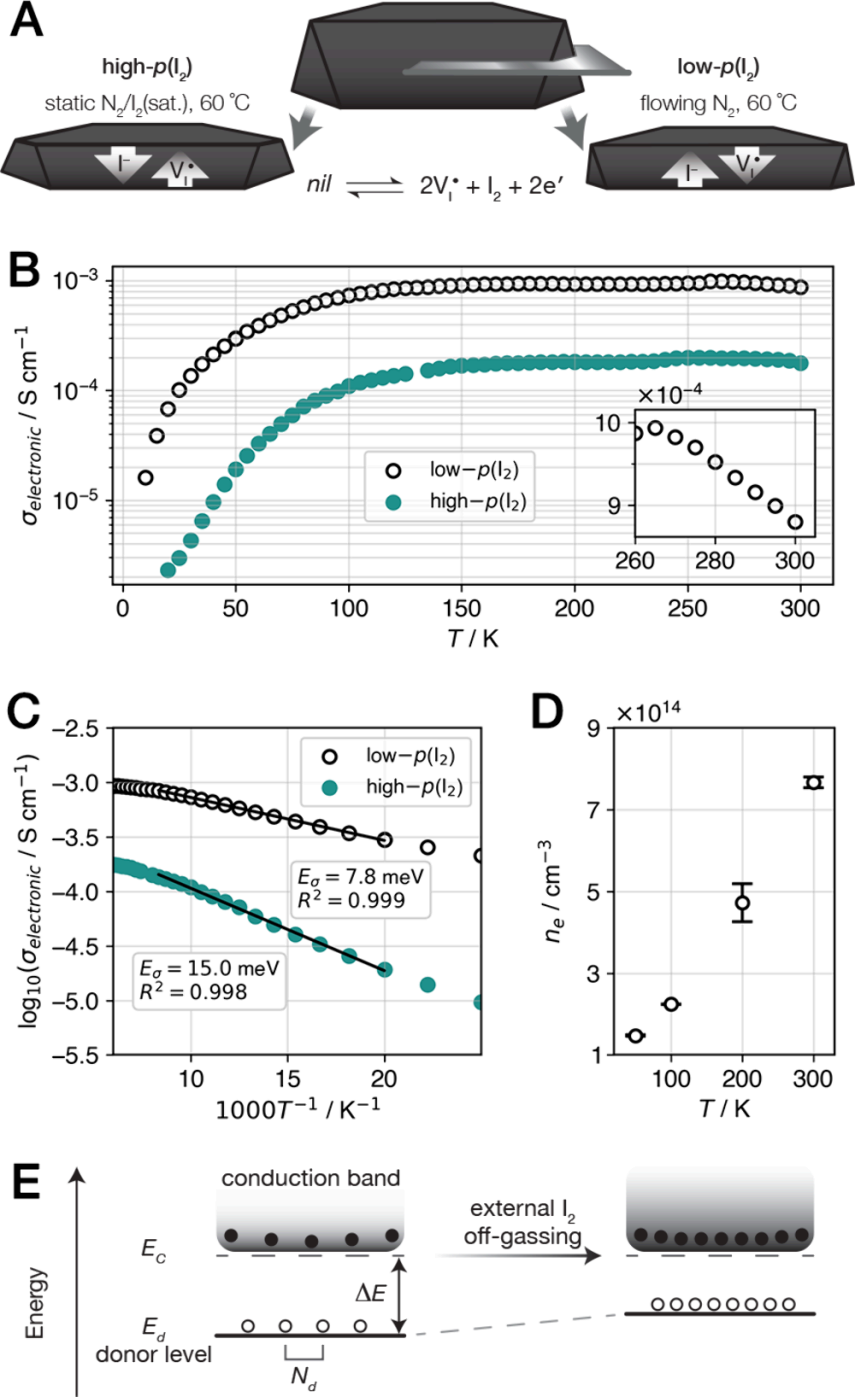
Electron transport properties of a Cs_2_SnI_6_ crystal at two points in the 2I^–^_(*s*)_/I_2(*g*)_ equilibrium. (A) Schematic representation of the treatment of the two halves of a Cs_2_SnI_6_ crystal for measurement with respect to the 2I^–^_(*s*)_/I_2(*g*)_ equilibrium. (B) Electronic conductivity measured for the low-*p*(I_2_) and high-*p*(I_2_) crystals; the degenerate semiconducting behavior of the low-*p*(I_2_) crystal near room temperature is shown in the inset. (C) Logarithmic plots of the conductivity and corresponding fits (activation energy, *E*_*σ*_, and statistics, *R*^2^, given) in the donor freeze-out region between 50 and 120 K. (D) Electron concentration (*n*_*e*_) quantified by the analysis of Hall effect measurements on the low-*p*(I_2_) crystal; error bars represent one standard deviation. (E) Schematic representation of the electronic energy levels near the conduction band of an *n*-type semiconductor. In Cs_2_SnI_6_, the iodine vacancy is the dominant donor defect and exhibits a small ionization energy, Δ*E* = *E*_*c*_ – *E*_*d*_, where *E*_*c*_ and *E*_*d*_ are the conduction band minimum and donor energy, respectively. Upon off-gassing, the increased density of iodine vacancies (*N*_*d*_) is accompanied by an increase in *n*_*e*_ and a shift in *E*_*d*_ toward the conduction band.

Comparative electron transport measurements were carried out on two thin Cs_2_SnI_6_ plates—cleaved from the same parent crystal—that were treated in opposite directions in the 2I^–^_(*s*)_/I_2(*g*)_ equilibrium (Figure 3A). One half of the crystal was exposed to the high-*p*(I_2_) treatment (see Model for Diffusion-Limited 2I^–^_(*s*)_/I_2__(*g*)_ Exchange Kinetics), whereas the other half was off-gassed under flowing N_2_ [denoted as low-*p*(I_2_)]. The phase purity of Cs_2_SnI_6_ was confirmed after each treatment by X-ray diffraction (Figure S8, Supporting Information), ruling out decomposition in the presence of I_2_ vapor as observed in (CH_3_NH_3_)PbI_3_ films.^52^ The details of these variable-*p*(I_2_) treatments and electron transport measurements are described in Methods.

We observed excellent agreement in the temperature dependence of the electronic conductivity between the two halves of the crystal (Figure 3B). The vertical offset of approximately one order of magnitude confirms that the electronic equilibrium is influenced from the same starting point by manipulating the external *p*(I_2_). Overall, the conductivity exhibits the expected temperature dependence of a semiconductor with uniform doping. The conductivity saturates at intermediate temperatures (150–300 K or *k*_*B*_*T* = 13–26 meV) below that required for intrinsic carrier generation (i.e., for valence-band electrons to be promoted across the band gap of 1.3–1.6 eV^43−45^ to the conduction band), indicating that charge carriers (electrons) from ionized donor defects are occupying the conduction band. Below ca. 125 K, as the temperature decreases toward—and below—the ionization energy of the donor defect, we observe donor freeze-out, where the donor defects remain neutral.

Interestingly, Cs_2_SnI_6_ also exhibits degenerate (heavily doped) semiconducting behavior near room temperature, as evidenced by decreasing electronic conductivity with increasing temperature (Figure 3B, inset). Metallic charge transport has been observed in the Sn^2+^ perovskites: (CH_3_NH_3_)SnI_3_ and CsSnI_3_.^53−55^ In these cases, hole concentrations can be large (10^19^ cm^–3^) owing to the presence of Sn^4+^ centers, which act as electron acceptors and lead to degenerate doping as the Fermi level dips below the valence band maximum. In contrast, Cs_2_SnI_6_ is redox-stable, *n*-type with a modest electron concentration (10^15^ cm^–3^), and exhibits metal-like charge transport, albeit in a very narrow temperature range. To the best of our knowledge, this is the first measurement of degenerate semiconducting charge transport in Cs_2_SnI_6_ (see Supporting Discussion S.D.3).

The logarithmic representation of the temperature-dependent conductivity further evidences a single linear region in the donor freeze-out regime (Figure 3C) that corresponds to the predominant electron donor in *n*-type Cs_2_SnI_6_. We attribute the observed donor freeze-out—with an onset temperature of ca. 125 K on cooling—to the iodine vacancy defect, and the slope informs on the vacancy donor ionization energy. The 2I^–^_(*s*)_/I_2(*g*)_ equilibrium (eq 2) can therefore be represented to consider the production of a charge-neutral iodine vacancy (*V*_*I*_^*x*^),
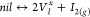
4followed by ionization,
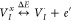
5where Δ*E* is the ionization energy. The electron-donor Δ*E* corresponds to the difference between the energy of the vacancy donor (*E*_*d*_) and the energy of the conduction band minimum (*E*_*c*_), shown schematically in Figure 3E. The activation energy derived from fits of the conductivity data (*E*_*σ*_ in log(σ_electronic_) ∝ −(*E*_*σ*_/*k*_*B*_) (*T*^–1^); Figure 3C) quantifies the thermal promotion of electrons from the iodine vacancy through *mE*_*σ*_ = Δ*E* (*m* = 1–2, depending on the level of doping and compensation in the crystal;^56^ the extent and consequences of compensation in Cs_2_SnI_6_ are addressed in detail in Supporting Discussion S.D.3). Thus, the fit values of 7.8–15 meV for *E*_*σ*_ suggest that *E*_*d*_ lies less than 30 meV below *E*_*c*_. These Δ*E* values also agree with the temperature dependence of *n*_*e*_ (via direct Hall effect measurement) in the same temperature regime (Figure S10 in the Supporting Information).

The electron mobility (*μ*_*e*_) and concentration allow us to rationalize the temperature dependence of the conductivity. The sign of the Hall voltage confirmed that Cs_2_SnI_6_ is an *n*-type conductor (Figure S9, Supporting Information). Upon cooling from 300 to 100 K, *μ*_*e*_ increases by a factor of ca. 3 (Figure S10, Supporting Information), consistent with various halide perovskite semiconductors exhibiting carrier mobility limited by optical phonon scattering.^57,58^ Below 100 K, the turnover and decreasing *μ*_*e*_ are characteristic of scattering from ionized defects.^57−59^ The electron concentration (*n*_*e*_) decreases monotonically with a shallow slope upon cooling from 300 to 50 K (Figure 3D). The thermal promotion of free electrons through the temperature range where the conductivity saturates (and, notably, down to 50 K) evidences the presence of a shallow donor defect. Taken together with the decreasing phonon population upon cooling, the electron mobility is likely limited by scattering off donor defects that remain ionized below 100 K.

Related observations of degenerate doping and a high fraction of ionized donors further support that both *E*_*d*_ and the Fermi level are proximal to *E*_*C*_ and shift closer upon external off-gassing (Figure 3E; see Supporting Discussion S.D.3). The decrease in Δ*E* upon self-doping (and, more generally, increased donor/acceptor doping and compensation) is an expected result from Coulomb interactions between ionized defects and carriers,^60−62^ particularly considering limited charge screening in Cs_2_SnI_6_ evidenced by a small optical dielectric constant.^44,63^ We can thus conclude that off-gassing (forward reaction, eq 2) self-dopes Cs_2_SnI_6_, given that we have shown that the iodine vacancy is both the majority defect and a shallow electron donor.

### Quantifying the Spontaneity of the Off-Gassing Reaction

**Figure 4:**
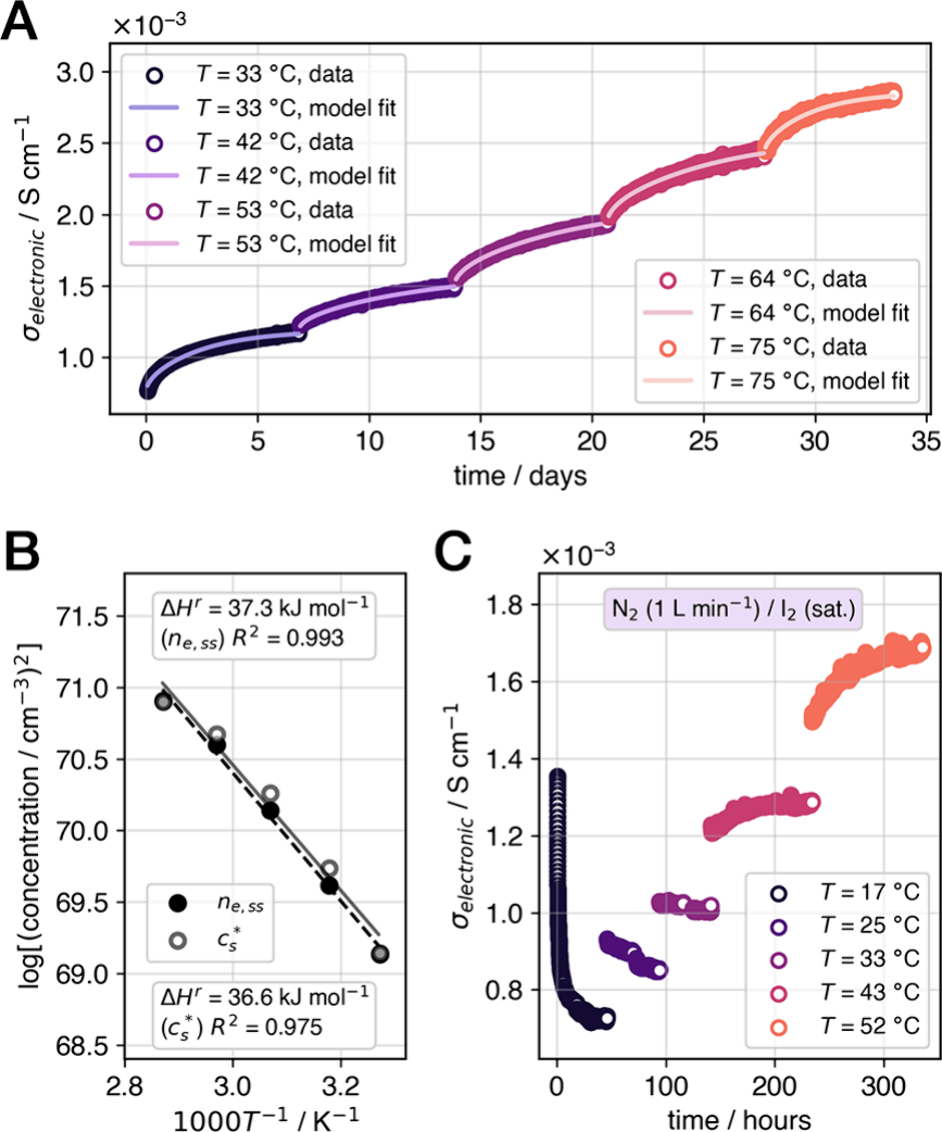
Thermodynamic analysis of the off-gassing reaction and defect modeling as a function of I_2_ partial pressure. (A) Temperature-step off-gassing conductivity measurements on a cleaved Cs_2_SnI_6_ crystal and model fits (main text, eq 3) under flowing N_2_ (3 L min^–1^). (B) Van ’t Hoff logarithmic plot of steady-state parameters from the off-gassing measurements that approximate the equilibrium electron concentration (*n*_*e,ss*_) and equilibrium excess vacancy concentration (*c*_s_*) and the corresponding fits (main text, eq 7; net enthalpy change Δ*H*^*r*^). (C) Temperature-step conductivity measurements on a cleaved Cs_2_SnI_6_ crystal under flowing N_2_/I_2_ (1 L min^–1^), where the I_2_ source was held at room temperature while the temperature of the measurement chamber (*T*) was increased in steps from 17 to 52 °C. Note that the slope changes sign between *T* = 33 and 43 °C.

Isothermal off-gassing conductivity measurements at five points above room temperature (Figure 4A) allow for the estimation of thermodynamic quantities associated with the equilibrium. The mass action—or equilibrium constant—expression for iodine exchange (eq 2) can be formulated in terms of the net free-energy change (Δ*G*^*r*^),
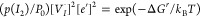
6where *P*_0_ is the standard pressure, [*V*_*I*_^·^] is the ionized monocationic iodine vacancy concentration, and [*e*′] is the electron concentration. The equilibrium electron concentration for a closed system is best approximated by the measured steady-state conductivity (*σ*_*ss*_ ∝ *n*_*e,ss*_ ≈ [*e*′]) and refined *c*_s_* values (*c*_s_* ≈ [*e*′]) from the diffusion model (eq 3). The diffusion model fits are shown in Figure 4A, and the corresponding optimized parameter values are given in Table S2. A logarithmic plot of the square of these steady-state concentrations is linear in *T*^–1^ (Figure 4B), as expected when eq 6 is transformed according to the Van ’t Hoff form,
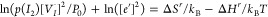
7where Δ*H*^*r*^ and Δ*S*^*r*^ are the net enthalpy and entropy changes, respectively. The fits are in good agreement in the estimation of Δ*H*^*r*^ (Figure 4B), yielding an average value of 37 kJ mol^–1^, or 0.38 eV.

The off-gassing reaction (forward reaction, eq 2) is thus endothermic; however, the gaseous product I_2_ indicates a significant entropic gain. The observation of spontaneous (Δ*G*^*r*^ < 0) off-gassing at room temperature confirms this and suggests that the corresponding Δ*S*^*r*^ is therefore greater than 0.12 kJ mol^–1^ K^–1^ (1.3 meV K^–1^) to overcome the enthalpic barrier. Indeed, we observed further evidence of the balance between entropic and enthalpic contributions under an I_2_-rich atmosphere (Figure 4C; see Methods). With a constant external *p*(I_2_), the electronic equilibrium shifts to the left as expected when the temperature of the crystal is below 33 °C. However, when the temperature increases to 43 °C and above, the conductivity derivative (with respect to time) changes sign. Thus, even under an external *p*(I_2_), the enthalpic barrier is overcome under moderate heating.

## Discussion and Outlook

We characterized spontaneous self-doping through the iodide–iodine exchange equilibrium in Cs_2_SnI_6_, an iodide double perovskite semiconductor. This external defect reaction—mediated by iodine vacancies—is analogous to the well-studied oxygen exchange reaction in oxide crystals. In oxides, off-gassing (forward reaction, eq 1) proceeds appreciably only at high temperatures, owing to the large, positive enthalpy of reaction. For instance, in samarium-doped CeO_2_, the enthalpy is ca. 4 eV per mole of O_2_ and off-gassing is therefore disfavored at room temperature despite a favorable entropy of reaction of ca. 1.1 meV K^–1^.^64^ Here, we observe a similar entropy of reaction and estimate the lower limit to be 1.3 meV K^–1^—which reflects the general nature of the solid–gas reaction—and a markedly lower enthalpy of 0.38 eV per mole of I_2_. A comprehensive comparison of thermodynamic quantities between the oxides and Cs_2_SnI_6_ is presented in the Supporting Information (Supporting Discussion S.D.4). Spontaneous iodine exchange and the related self-doping are likely general to iodide perovskites owing to the small enthalpy of reaction and the similarity among metal–iodide bond enthalpies across main-group B-site metals such as Pb and Sn.^25^

**Figure 5:**
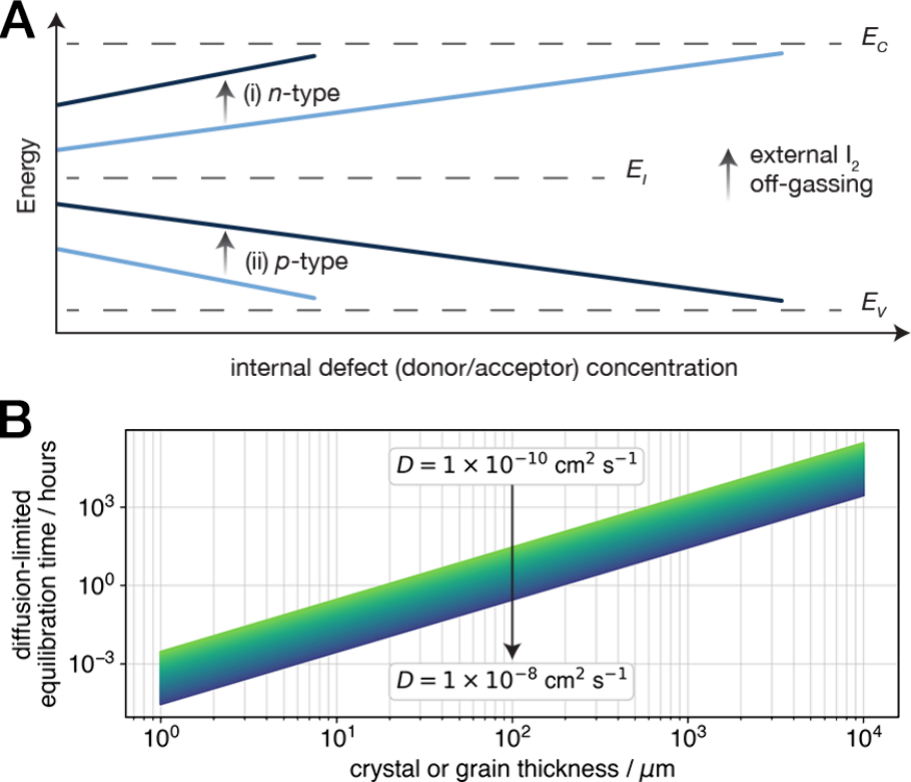
Implications of the exchange equilibrium on perovskite semiconductors. External iodine (I_2_) off-gassing shifts the room-temperature Fermi level (solid lines, A) in various iodide perovskite semiconductors through the generation of excess iodine vacancy donors, resulting in (*i*) a self-doping effect on *n*-type perovskites, as in Cs_2_SnI_6_ and (*ii*) a self-compensation effect on *p*-type perovskites such as (CH_3_NH_3_)SnI_3_. The external exchange equilibrium therefore augments the typical control exerted over electronic properties through internal stoichiometry and dopant control (*x* axis, A). Vacancy defect generation simultaneously increases the bulk ionic conductivity, and the one-dimensional diffusion model applied here allows for the prediction of the equilibration time for off-gassing from a crystal or grain of arbitrary thickness, provided that the off-gassing kinetics are diffusion-limited within the crystal (B).

External *p*(I_2_) can have a similarly drastic effect on the properties of iodide perovskites beyond Cs_2_SnI_6_, even if the doping (*n* or *p* doping) or defect levels differ. It follows that an appreciation for this external defect reaction will promote our capacity to understand—and predict—the extent to which spontaneous off-gassing may influence electronic properties (Figure 5A). One such example studied elsewhere is polycrystalline *p*-type (CH_3_NH_3_)PbI_3_,^4,14,40^ where a shift in the work function was observed under an I_2_ atmosphere.^40^ The authors attributed the shift in Fermi level—further toward the valence band—to a reduction in iodine vacancy donors (*V*_*I*_; reverse reaction, eq 2) based on the low vacancy formation energy (compared to that of iodine interstitials). Off-gassing would therefore drive (CH_3_NH_3_)PbI_3_ toward the intrinsic limit by a compensation effect, reducing the majority carrier (hole) concentration and shifting the Fermi level toward the conduction band. In summary, spontaneous off-gassing may manifest in various ways: (*i*) a self-*n*-doping mechanism shifting the Fermi level ever closer to the conduction band (e.g., Cs_2_SnI_6_; Figures 3E and 5A); (*ii*) an external compensation mechanism for *p*-doped perovskites [e.g., (CH_3_NH_3_)SnI_3_; Figure 5B] that reduces the hole concentration; and (*iii*) a gradual *p*- to *n*-type transition in lightly doped perovskites [e.g., (CH_3_NH_3_)PbI_3_].

Finally, we note that spontaneous off-gassing (forward reaction, eq 2) from a polycrystalline thin film is likely detrimental to the stability of a perovskite-based device owing to the stoichiometric production of corrosive I_2_ and excess iodine vacancies. The generation of gaseous I_2_ in a confined device stack presents multiple potential failure mechanisms including reactions with metallic contacts. Meanwhile, iodine vacancies produced after film deposition counteract the observed benefits of vacancy-filling additives^65,66^ by increasing the bulk ionic conductivity and restoring undesired ionic polarization effects.

The diffusion model we apply here may be instructive for determining characteristic time scales of diffusion-limited iodide–iodine exchange for a particular crystal or grain size in a polycrystalline film (Figure 5B). Indeed, recent work demonstrates that halide diffusion in the volume of perovskite grains is orders of magnitude slower than grain boundary diffusion (and hence bulk-diffusion-limited) in various 3D perovskite thin films.^67^ A complete picture of polycrystal grain sizes and relative contributions of transport along and within grains, when combined with surface treatments to influence exchange rates,^68^ presents promising avenues for kinetic stabilization. At long times, however, the equilibrium vacancy concentration is thermodynamically controlled, and shifting the equilibrium constant of the iodide–iodine exchange reaction may require compositional tuning or I_2_-impermeable device encapsulation layers that maintain local saturation of I_2_ to disfavor off-gassing.

Altogether, the insights we derive from the reversibility, kinetics, and thermodynamics of the iodide–iodine exchange defect reaction in Cs_2_SnI_6_ are likely general to the broad family of halide perovskites, importantly including compositions heavily investigated as photovoltaic absorbers. The prevalence of *X*_2_ off-gassing thus calls for re-evaluation of the characteristic defect reactions, defect-mitigating treatments, and encapsulation materials to obtain stable optoelectronic properties from halide perovskite semiconductors. This is particularly important for thin-film photovoltaic modules, where the surface area of the absorber—where the halide–halogen exchange reaction occurs—is significant.

## Methods

### General Methods

All manipulations were performed in an ambient atmosphere and at room temperature unless otherwise noted. All reagent-grade or higher-purity chemicals were purchased from commercial vendors and used as received unless otherwise noted.

### Safety Statement

No unexpected or unusually high safety hazards were encountered.

### Synthesis and Recrystallization of SnI_4_

Solid I_2_ (28.3 g, 112 mmol) and Sn^0^ powder (7.61 g, 64.0 mmol) were combined in 75 mL of chloroform and heated at reflux near 60 °C with stirring. When the solution turned colorless after about 24 h, the crude product was isolated by rotary evaporation under reduced pressure. Solid SnI_4_ (34 g, 97% yield) was recrystallized from the crude product by sublimation–recrystallization at 80 °C and under dynamic vacuum.

### Growth of Cs_2_SnI_6_ Crystals

Stoichiometric precursor solutions were prepared by dissolving CsI (520 mg, 2.00 mmol) and SnI_4_ (626 mg, 1.00 mmol) in 10 mL of dry γ-butyrolactone (stored over 3 Å molecular sieves) at 130 °C. The solution was cooled slowly from 130 °C to room temperature at 1–2 °C h^–1^ and subsequently allowed to rest undisturbed for several days. A typical crystallization produced several large (2–8 mm edge length) crystals and numerous small crystals, with most exhibiting a truncated octahedral habit. The Cs_2_SnI_6_ crystals were stored in the dark in a sealed vial in the mother liquor prior to preparation for measurements.

### Preparation of Crystals for Conductivity Measurements

Large Cs_2_SnI_6_ crystals (>4 mm edge length, exemplified in Figure 1C, main text) were isolated from the mother liquor and immediately cleaved parallel to the largest exposed plane (typically a {111} termination) with a razor blade. If necessary, the crystal surface was dry-polished with 5 μm and 1 μm alumina polishing paper, and the resulting powder was removed from the surface with a stream of dry N_2_. For all electron transport measurements, the thickness was reduced to less than 1 mm. The crystal was then mounted on a sapphire wafer and fixed in place using double-sided Kapton tape or poly(methyl methacrylate) (PMMA; from a solution in toluene, 0.13 g mL^–1^). For ionic conductivity measurements, the thickness was reduced to produce equally sized parallel exposed planes for contact with the AgI solid electrolyte (see below). All transport measurements took place in the dark and under an N_2_ atmosphere, unless otherwise noted.

### Electronic Conductivity Measurements

Graphite contacts were applied to the surface of the crystal via a toluene suspension (ca. 100 mg mL^–1^) to form four small, equally spaced contact pads along the edge of the surface for four-point measurements. The surface area of the contact pads was minimized with respect to the intercontact distance on the surface of the crystal, per the van der Pauw methodology.^69^ The graphite pads were contacted with graphite-coated spring steel fingers connected to external leads passing through the walls of a custom-built measurement chamber. A representative Cs_2_SnI_6_ crystal contacted as described is shown in Figure 1E (main text). The walls of the chamber were stainless steel; all measurements were therefore carried out in the dark.

The temperature was monitored within the sample chamber by using a glass-encapsulated thermistor (YSI) placed adjacent to the crystal on the measurement block. The chamber remained closed to the ambient atmosphere and under positive pressure of inert N_2_ gas (99.998%) throughout the measurement; a flow meter (Dwyer) was used to modulate the rate of N_2_ flow. The N_2_ gas passed through a packed bed with heated lab armor beads, was monitored with a K-type thermocouple, and was adjusted to match the temperature of the thermistor within the chamber. The external temperature of the measurement chamber was controlled by partially submerging the chamber in a temperature-controlled sand bath.

Four-point electronic conductivity measurements were made in the van der Pauw geometry using a BioLogic VSP-300 potentiostat/galvanostat. The voltage channels (V^+^, V^–^) and the electrical source channels (I^+^, I^–^) were configured in four possible configurations with orthogonal geometry (P_0_, *P*_90_, *P*_180_, and *P*_270_; Figure S11A, Supporting Information). Individual contact resistances were first confirmed by inspection of the low-frequency limit of potentiostatic electrochemical impedance spectroscopy (PEIS) measurements. A typical PEIS measurement utilized a 26 mV RMS amplitude, starting at 1 MHz and ending at 100 mHz. Galvanostatic electrochemical impedance spectra (GEIS), typically with an amplitude of between 0.5 and 2 μA, were then measured in the same frequency range (1 MHz to 100 mHz) on individual paths to confirm that equivalent configuration pairs (P_0_/*P*_180_, *P*_90_/*P*_270_) were in sufficient agreement for averaging. The resistance (*R*) was then calculated from the resistance values at the DC limit (in this case, 100 mHz) for equivalent configuration pairs,
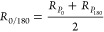
8
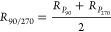
9and the electronic conductivity (σ) was subsequently calculated,
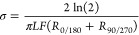
10where *L* is the thickness of the crystal and *F* is the van der Pauw factor.^69^ A typical GEIS measurement is shown in Figure S1, exhibiting a fast approach to the DC limit that motivated the use of a 100 mHz frequency for single-point GEIS measurements in the approach that follows.

### Continuous Off-Gassing Electronic Conductivity Measurements

For continuous electronic conductivity measurements, as in the temperature-step off-gassing traces (Figures 2D and 4A, main text), a multichannel measurement on adjacent paths (Figure S11A, Supporting Information) was implemented to allow for the simultaneous determination of *F*. Here, identical single-point GEIS measurements (at a frequency of 100 mHz) were synchronized to alternate in time to measure one of two orthogonal paths, for instance, *P*_0_ and *P*_90_. In this case, the electronic conductivity takes the alternative form,
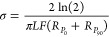
11due simply to the lack of averaging between equivalent paths. A single measurement of all paths before and after the continuous measurement was used as confirmation that the equivalent configuration pairs (*P*_0_/*P*_180_, *P*_90_/*P*_270_) remained in sufficient agreement to justify the simplification from eq 10 to eq 11.

Continuous temperature-step off-gassing measurements were carried out under a 3 L min^–1^ N_2_ flow in the closed measurement chamber. After contacting the crystal and sealing the chamber, the system was typically allowed to equilibrate at room temperature for at least 7 days. Subsequent isothermal measurements were spaced by 10–15 °C and continued for 5 to 7 days at each temperature. The temperature jump (in the chamber and the flow, see above) produced a small offset in the conductivity within the first hour of the measurement. As the long-time isothermal behavior was of interest in the analysis, the fast thermal equilibration was omitted from analysis and model fitting (see below).

### In-Line I_2_-Dosing Electronic Conductivity Measurements

During a typical continuous, isothermal electronic conductivity measurement—as described above—under flowing N_2_ (1–1.5 L min^–1^), a column of solid I_2_ (5–8 in. tall) was introduced upstream from the measurement chamber. The column was maintained at room temperature, providing a continuous flow of N_2_/I_2_ vapor near the saturated vapor pressure of I_2_ at this temperature (ca. 40.6 Pa^47^). With independent control of the measurement chamber, the crystal was either maintained at the temperature prior to the introduction of I_2_ (exemplified in Figure 2A, main text) or increased in increments of 8–10 °C (exemplified in Figure 4C, main text). The outgoing flow from the measurement chamber passed through a cold trap (dry ice in isopropyl alcohol) to recover solid I_2_ and a subsequent aqueous solution of KI to scrub remaining I_2_ from the efflux.

### Ionic Conductivity and Diffusion Measurements

Polycrystalline AgI pellets (circular; 13 mm diameter) were prepared in a dry pellet die (Carver) under 6000 pounds of pressure in a nitrogen-filled glovebox. The AgI pellets acted as a solid electrolyte and as the electron-blocking electrode to contact a cleaved Cs_2_SnI_6_ crystal in a symmetrical cell: Ag|AgI|Cs_2_SnI_6_|AgI|Ag. The Ag paint (Ted Pella) served as the contact between AgI and the external cell leads.

Two-point ionic polarization and conductivity measurements were made by using a BioLogic VSP-300 potentiostat/galvanostat. The above cell was polarized at room temperature by applying a constant current (*I*) of 1 nA and monitoring the potential drop (*E*) across the cell, which is dominated by the ionic resistivity of Cs_2_SnI_6_ owing to the high ionic conductivity of AgI. The ionic conductivity (σ) was calculated using an appropriate relation for the two-point geometry,
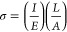
12where *A* is the average contact area of the surface facets of the crystal.

Following the polarization period, the applied current was removed, and the open circuit potential of the cell was monitored for 24 h. The potential is expected to approach zero with a characteristic time corresponding to the diffusion coefficient of the dominant mobile ion and the thickness of the crystal.^46^ The dominant mobile ion and defect were determined to be the iodide anion and iodine vacancy, respectively, from a solid-state coulometry measurement.^31^ These measurements and analyses are described in detail in the Supporting Information (Supporting Methods and Figures S4 and S5).

### One-Dimensional Diffusion Model for Iodine Vacancy Transport

The solution to Fick’s Second Law for a flat plate gives a one-dimensional concentration profile in *x* of the form
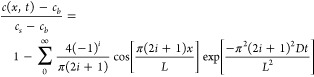
13where *c* is the excess defect concentration, *c*_*b*_ and *c*_*s*_ are the bulk and surface excess defect concentrations, respectively, and *x* is the out-of-plane dimension (Figure 2B, main text).^50,51^ The characteristic time for relaxation is defined as τ:
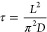
14

Integrating the excess defect concentration profile (eq 13) affords an analytical expression for the bulk electronic conductivity,
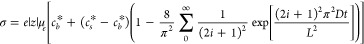
3awhere *e* is the elementary charge, *z* is the charge of the defect (1+), *μ*_*e*_ is the electron mobility, and the * superscript on the concentrations defined above reflect the assumption that the defects are ionized. In the long-time limit (*t > τ*), a power-law linearization that captures the same characteristic time dependence can be applied to simplify the functional form and provide a secondary determination of *D* (see Supporting Methods and Figure S12, Supporting Information).

The parameters *D*, *c*_*b*_, and *c*_*s*_ were varied to refine the model against experimental off-gassing electronic conductivity data (described above). The inputs for the initial guess, upper bound, and lower bound of *D*, *c*_*b*_, and *c*_*s*_ remained constant across all fits to reduce the bias. Known values of *e* and *z* and measured values of *L* and *μ*_*e*_ (7.17 cm^2^ V^–1^ s^–1^, at 300 K from Hall effect measurements) were treated as constants in the fitting. The regression was built from a least-squares minimization with respect to the full analytical expression (eq 3). The diffusion coefficient was confirmed by a subsequent linear regression of the power-law linearization (above). The measured data were fit only after the temperature within the chamber stabilized following a 10–15 °C temperature increase from the prior steady state. Each continuous isothermal data set was fit independently. Full data processing and model fitting were performed using a custom python code based on modules from the SciPy^70^ and Scikit-learn^71^ packages.

### Variable *p*(I_2_) Treatments

The Cs_2_SnI_6_ crystals were stored in a dark, dry atmosphere after isolation from the mother liquor or following a continuous conductivity measurement (see above). As described in the main text, crystals were subjected to distinct treatments to influence the iodine exchange equilibrium. A low-*p*(I_2_) treatment was designed to shift the equilibrium toward the forward reaction (eq 2); here, the crystal was transferred to a clean, dry vessel and held at a constant temperature (typically 60 °C) with a continuous flow of dry N_2_. Alternatively, a high-*p*(I_2_) treatment was designed to shift the equilibrium toward the reverse reaction. Here, a crystal was transferred to a small shell vial and placed within a larger vial with excess solid I_2_. The vials were flushed with dry N_2_ and sealed before being held at constant temperature (typically 60 °C, corresponding to a saturated I_2_ vapor pressure of >400 Pa^47^). In both cases, the condition was maintained for a time corresponding to at least 3τ (thus dictated by the size of the crystal) to allow for complete equilibration, which typically lasted more than 5 days. Following the treatment, the crystal was cleaved or polished to produce a clean surface prior to subsequent measurement.

### Electron Transport Measurements

A physical property measurement system (PPMS) from Quantum Design was equipped with a lock-in amplifier (SR860, Stanford Research Systems), a current/voltage source (Lakeshore MeasureReady 155 precision), a switchbox (7001 Keithley), and two switching cards (7012-S 4x10). A reported experimental design was followed for variable temperature conductivity and Hall effect measurements.^72^ By using a switch box, the voltage channels (V^+^, V^–^) and the electrical source channels (I^+^, I^–^) were placed in four different configurations (*P*_0_, *P*_90_, *P*_180_, and *P*_270_) for the conductivity measurements (Figure S11A, Supporting Information) or in the Hall effect configurations (H_0_^+^, H_0_^–^, H_90_^+^, and H_90_^–^) to elucidate the carrier type and concentration (Figure S11B, Supporting Information).

Conductivity measurements were performed at ca. 7 mTorr. The variable-temperature conductivity of the sample was determined by using the van der Pauw method by measuring the AC resistance in the four conductivity configurations at a current of 0.1 μA and a frequency of 11 Hz. The time constant for the measurement was 1 s, averaging over at least ten cycles for all frequencies and yielding stable voltage readings with four significant figures. After the voltage stabilized for the first reading, a standard wait time of 10 s was used before recording each subsequent voltage at different temperature set points. The phase of the AC measurements was monitored, and the phase shifts did not exceed 1°. The resistances of the equivalent configuration pairs were averaged (eqs 8 and 9), and the electronic conductivity was subsequently calculated (eq 10).

Hall effect measurements were performed for each electrode configuration separately at a current of 0.1 μA and a frequency of 21 Hz for the four Hall effect configurations (H_0_^+^, H_0_^–^, H_90_^+^, and H_90_) at 300 K and for the H_0_^+^ and H_90_^+^ configurations below 200 K. The applied magnetic field (*B*) varied between −3 and 3 T at a rate of 0.01 T s^–1^, and the electrical conductivity was measured every 0.25 T. The time constant for the measurement was 1 s, with an averaging time of 5 s. This time constant provided sufficient averaging such that noise was an insignificant source of error.

The Hall voltage was extracted from the data in the 3 *T* > *B* > −3 T region for each configuration. For the 300 K measurement, the zero-field voltage drift was subtracted from the measured voltage (see below). For all measurements, the electron concentration (*n*_*e*_) was calculated according to
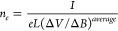
15where (Δ*V*/Δ*B*)^*average*^ is the Hall voltage divided by the magnetic field. The average quantity was determined from a linear regression of the experimental data collected by varying the magnetic field. The measurement error was estimated using the standard deviation between the (Δ*V*/Δ*B*) values from distinct configurations.

At 300 K, the sample resistance changed over time without the application of a magnetic field. We attribute this zero-field resistance drift to additional off-gassing caused by the reduced pressure in the PPMS. To mitigate the effect of the zero-field voltage drift in the Hall effect measurement at 300 K, we estimated its drift by subtracting (or adding) the slope of the voltage–time curves in the 3 *T* > *B* > −3 T region. This protocol is described in detail in a previous report.^72^ At 200 K and below, no correction was applied (no drift was observed).

Electron mobility was calculated according to
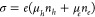
16where μ is the carrier mobility, *n* is the carrier concentration, and the indices indicate the carrier type (*e*, electron; *h*, hole). The hole concentration (*n*_*h*_) was assumed to be vanishingly small, such that *μ*_*h*_*n*_*h*_ ≪ *μ*_*e*_*n*_*e*_.

## Data Availability

The data that support the figures within this Article and code used to perform model fitting are available from the corresponding author upon request.

## Supporting Information Available

Supporting methods, Figures S1–S12, Tables S1–S6, Discussions S.D.1–S.D.4, and references (PDF)
Code used in diffusion model fitting (PDF)
