# Supplementary material for: Halide Perovskites Breathe Too: The Iodide–Iodine Equilibrium and Self-Doping in Cs2SnI6
Source: ACS Cent Sci. 2024 Apr 2;10(4):907–19. doi: 10.1021/acscentsci.4c00056 (PMC11046464; doi:10.1021/acscentsci.4c00056)
Supplement: Supplementary file 1 — oc4c00056_si_001.pdf [file oc4c00056_si_001.pdf]

Supporting Information

Halide perovskites breathe too: The iodide-iodine equilibrium and self-doping in Cs_2SnI_6

Julian A. Vigil,[†,‡] Nathan R. Wolf,[†] Adam H. Slavney,[†] Roc Matheu,[†] Abraham Saldivar Valdes,[†] Aaron Breidenbach,[§,ǁ] Young S. Lee,[¶,ǁ] and Hemamala I. Karunadasa[†,ǁ,*]

[†] Department of Chemistry, Stanford University, Stanford, California 94305, United States
[‡] Department of Chemical Engineering, Stanford University, Stanford, California 94305, United States
[§] Department of Physics, Stanford University, Stanford, California 94305, United States
[¶] Department of Applied Physics, Stanford University, Stanford, California 94305, United States
[ǁ] Stanford Institute for Materials and Energy Sciences, SLAC National Laboratory, Menlo Park CA 94025
*e-mail: hemamala@stanford.edu

Table of Contents:

Supporting Methods S2
Supporting Figures S6
Supporting Tables I S18
Supporting Discussion S20
Supporting Tables II S26
Supporting References S28

Supporting Methods

X-ray diffraction measurements. Diffraction patterns were collected on a Bruker D8 Advance diffractometer (Cu K_α radiation, $K_{\alpha,1}$:$K_{\alpha,2}$ ≈ 2:1; Bragg-Brentano θ -θ geometry) or through mail-in services at the Advanced Photon Source (APS; beamlines 11-BM and 11-ID-B) at Argonne National Laboratory. Powders of Cs_2SnI_6 were ground from bulk crystals using a mortar and pestle. For measurement in reflection (Bragg-Brentano) geometry on the Bruker D8 Advance, powders, and electrodes (from the coulometry experiment, see below) were mounted on a zero-background stage and single crystals were mounted on a clay holder. High-resolution X-ray diffraction measurements at beamline 11-BM of the APS were collected with 27-keV radiation (λ = 0.458 Å); neat powders were loaded into a Kapton capillary (ID = 0.8 mm) for measurement in transmission (Debye-Scherrer) geometry ($\mu \times$ r = 1.98). Low-resolution X-ray scattering measurements at beamline 11-ID-B of the APS were collected with 87-keV radiation (λ = 0.143 Å); neat powders were loaded into a Kapton capillary (ID = 0.8 mm) for measurement in transmission geometry ($\mu \times$ r = 0.5). Data from 11-ID-B were reduced with custom python code using the pyFAI[1] package. Diffraction patterns are represented in the main text and **Figure S8** as a function of the scattering vector, q, which is related to the wavelength and scattering angle (θ) through $q = (4\pi/\lambda)\sin(\theta)$. Lab-source data converted from θ to q used the wavelength of Cu $K_{\alpha,1}$ radiation owing to the negligible contribution of $K_{\alpha,1}/K_{\alpha,2}$ splitting in the angle range of interest (as in "cleaved Cs_2SnI_6 crystal", Figure 1D, main text).

Open circuit potential relaxation following stoichiometric polarization. The diffusion coefficient was determined from the open circuit potential by analyzing the long-time relaxation behavior after an extended period of polarization. Here, 1 nA direct current was applied to a symmetric AgI | Cs_2SnI_6 | AgI cell (see Figure 1F and Methods, main text) for ~17 h (**Figure S2A**). The voltage across the cell reached a nearly constant steady-state potential (E_{ss}) of ~0.3 V, indicative of the ionic resistivity of Cs_2SnI_6. Upon removal of the applied current, the open circuit potential (E) decreases rapidly before relaxing more slowly after approximately 1 h (**Figure S4A**). At short times (t), the relaxation exhibits $(t)^{1/2}$ dependency; at longer times, the open circuit potential follows a power law of the following form:

$$E = E_{ss}e^{-\frac{\pi^2 Dt}{L^2}}$$

Thus, a corresponding linearization of the difference between E_{ss} and E at time t,

$$\log\left(E_{ss} - E\right) \propto \frac{\pi^2 Dt}{L^2}$$

allows for determination of the diffusion coefficient (D) from the fitted slope (**Figure S4B** and Figure 1H, main text) and the thickness (L) of the crystal. A bulk single crystal (L = 0.46 mm) of Cs_2SnI_6 was used for the measurement, thus the fit was constrained to the last 4 h to ensure the appropriate application of the long-time power-law linearization. Values of D determined from fitting of the short time behavior agreed with the linear fits within approximately an order of magnitude (in units of $cm^2\ s^{-1}$). However, here

(and below, in the context of the diffusion model linearization) we emphasize that quantification of D tends to be more reliable in the long-time limit owing to competing processes at short times, including temperature fluctuation, interfacial equilibration, and space-charge redistribution.[2]

Simulating transient components of ionic and electronic polarization. Selective electrodes produce stoichiometry polarization effects in materials during (and after) polarization, particularly for mixed ionic-electronic conductors such as $(CH_3NH_3)PbI_3$.[3] The transient response to galvanostatic load can be simulated for a general case using only the ionic and electronic conductivity of the contacted material.[4] Here, we simulated the response of Cs_2SnI_6 in a symmetric cell with ideal ion-blocking, electron-conducting electrodes under 1 μA of applied direct current (**Figure S3**).

The ionic and electronic components of the current (I_{ionic} and $I_{electronic}$, respectively) can be derived from an equivalent circuit representation, or by summing the dominant resistances with a simplifying assumption that the timescale of stoichiometry polarization is larger than that of interfacial processes.[4] The relevant expressions—for ion-blocking, electron-conducting electrodes—describing the switch-on behavior from open circuit (to galvanostatic polarization),

$$\mathrm{I_{ionic}} = \frac{R_{electronic}}{R_{ionic} + R_{electronic}} I_{app} \mathrm{e}^{-t/\tau}$$

$$\mathrm{I_{electronic}} = I_{app} \left(1 - \frac{R_{electronic}}{R_{ionic} + R_{electronic}}\right) \mathrm{e}^{-t/\tau}$$

as well as the switch-off behavior after polarization (to open circuit),

$$\mathrm{I_{ionic}} = -\frac{R_{electronic}}{R_{ionic} + R_{electronic}} I_{app} \mathrm{e}^{-t/\tau}$$

$$\mathrm{I_{electronic}} = I_{app} \left(\frac{R_{electronic}}{R_{ionic} + R_{electronic}} - 1\right) \mathrm{e}^{-t/\tau}$$

depend on the applied current (I_{app}), the bulk ionic and electronic resistivity (R_{ionic} and $R_{electronic}$, respectively), and the timescale of stoichiometry polarization (τ). The observed potential during polarization, E, is related to electronic components $R_{electronic}$ and $I_{electronic}$ through Ohm's law, $E = IR$.

Stoichiometry polarization refers to the combination of chemical diffusion and drift processes that produce a net stoichiometry change (or gradient) in an ionic material. In the context of ion-blocking electrodes, the ionic flux (j_{ion}) is zero; thus, stoichiometry polarization here refers to the requirement of an internal ion gradient ($\nabla\mu_{ion}$) to balance the applied potential ($\nabla\phi$) and satisfy the flux condition,

$$\mathrm{j_{ion}} = 0 \ \therefore \ \nabla\bar{\mu}_{ion} = \nabla\mu_{ion} + z_{ion}\nabla\phi = 0$$

where $\nabla\bar{\mu}_{ion}$ and z_{ion} are the electrochemical potential and charge of the mobile ion.

Coulometric reaction cell experiment and characterization. Solid-state coulometry was used to identify the dominant mobile ion in Cs_2SnI_6. The principles behind this class of polarization experiments, sometimes referred to as "reaction cells", have been described in detail elsewhere.[3, 5, 6] Coulometry experiments have been applied to perovskites since the 1980s to determine the dominant mobile ion.[7] Briefly, application of direct current to an asymmetric cell induces an interfacial reaction at one side of the conductor (here, Cs_2SnI_6) based on the directionality of the field and the corresponding drift of the dominant mobile ion. We used X-ray diffraction (see above) to characterize the interfaces between Cs_2SnI_6 and the electrodes to identify crystalline reaction products.

Individual pellets of polycrystalline Cs_2SnI_6 and AgI (circular; 13-mm diameter) were prepared from bulk powders in a dry pellet die (Carver) under 6000 pounds of pressure in a nitrogen-filled glovebox. Powders of Cs_2SnI_6 were ground from bulk crystals using a mortar and pestle. An asymmetric cell featuring AgI and Pb contacts was constructed to assess transport and interfacial reactivity under polarization, specifically Ag | AgI | Cs_2SnI_6 | Pb | Ag (**Figure S5A**). Prior to cell construction, Pb foil was polished with an abrasive to remove an apparent oxide layer at the surface of the metal. Ag paint (Ted Pella) served as the contact between AgI (or Pb) and the external cell leads. The cell was transferred to a N_2 atmosphere with temperature control (see Methods, main text, for details of the custom chamber) and 100 nA direct current was applied to the cell at 50 °C for 7 days. The cell was deconstructed and the Pb | Ag electrode interfaces were examined by X-ray diffraction (**Figure S5B**). An identical cell was constructed and allowed to rest at open circuit potential for 7 days to confirm that spontaneous reactivity was not observed in the absence of applied current.

Linearization fitting within the one-dimensional diffusion model formalism. The details of the full one-dimensional diffusion model applied to the transport of iodine vacancies are given in the main text (Methods). In a similar manner to the stoichiometry polarization response (see above), an off-gassing conductivity measurement is diffusion-limited and thus exhibits characteristic time-dependence related to the chemical diffusion coefficient *D*. In this case, the analytical expression for the diffusion model (Eq. 3, main text) is used to obtain a simplified power law involving a dimensionless conductivity factor (defined as M_σ),[2] given that the first term in the exponential (of Eq. 3, main text) will dominate as *t* approaches τ:

$$\frac{\sigma_{electronic} - e|z|\mu_e c_b^*}{e|z|\mu_e(c_s^* - c_b^*)} = \frac{8}{\pi^2}e^{-t/\tau}$$

The constant τ is defined in the main text Methods (Eq. 14) and is typically in the range of 15-100 h for off-gassing measurements on Cs_2SnI_6 (see, for instance, **Table S1** and **S3**). Here, c_b^* and c_s^* are the bulk and surface ionized excess defect concentrations, *e* is the elementary charge, *z* is the charge of the defect (1+), μ_e is the electron mobility (see Methods, main text). Thus, fitting of the linearized power law,

$$\ln(M_\sigma) = \ln\left(\frac{8}{\pi^2}\right) - \frac{\pi^2 Dt}{L^2}$$

with a fixed intercept yields D (**Figure S12**). The estimate of D obtained using this method agrees well with the full model fitting described in the main text, though we note they are inherently related through the inputs c_b^* and c_s^*. During a typical fitting, the full model fitting was performed as described in the main text and the fit values for c_b^* and c_s^* were subsequently used to re-optimize the model fit and perform the power law linearization. The reported values for D (**Table S1** and **S3**) are an average of the estimates obtained from the two methods.

Supporting Figures

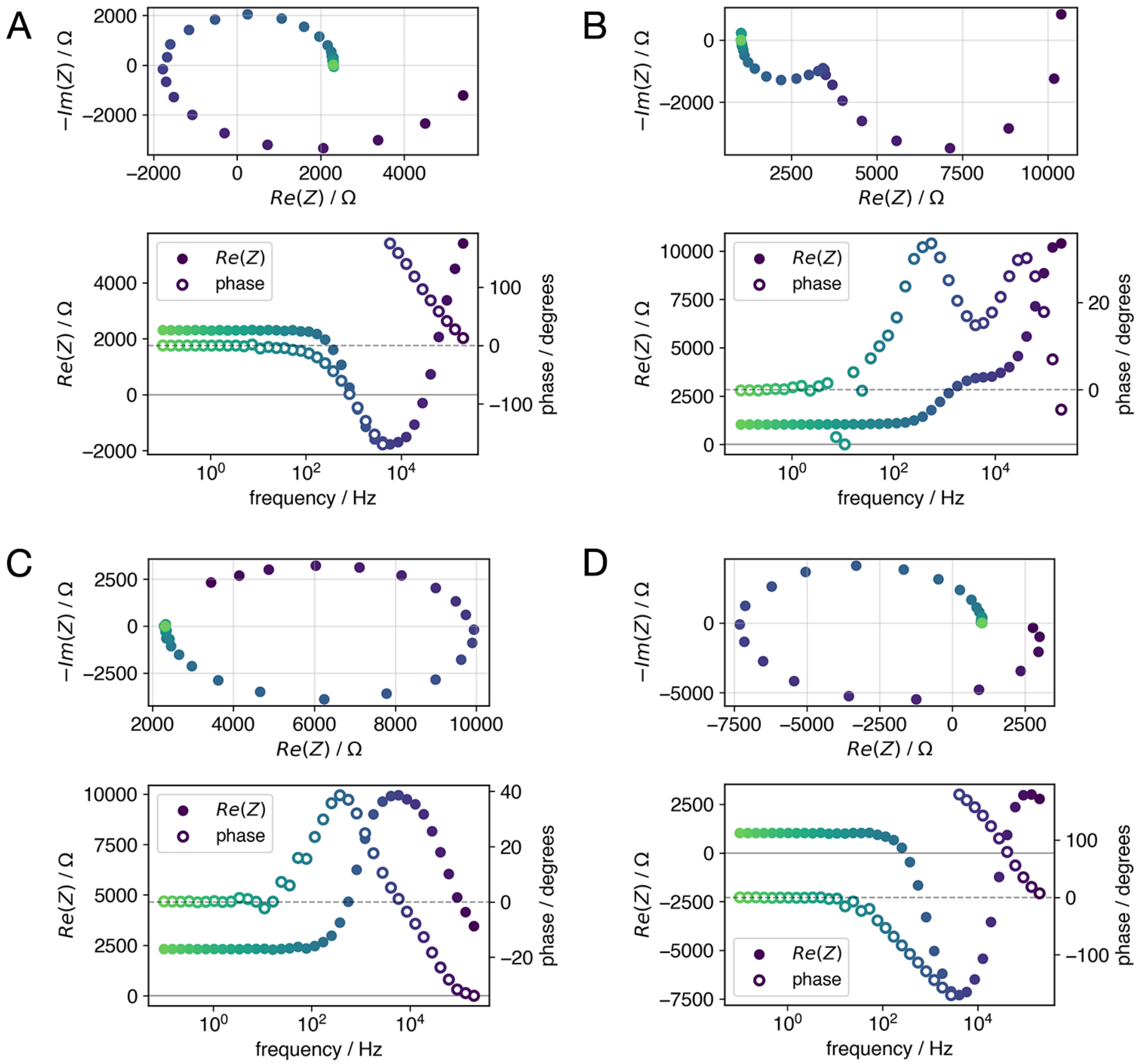

Figure S1. Galvanostatic impedance spectroscopy measurements on each of the four configurations (**A**-**D**) of a representative Cs_2SnI_6 crystal (thickness = 0.68 mm) contacted in the van der Pauw geometry (see Figure 1E, main text). The top panels show the Nyquist representation of the impedance spectrum with the real [*Re*(*Z*)] and imaginary [*Im*(*Z*)] components of the impedance. The bottom panels show *Re*(*Z*) and the phase angle as function of frequency. Upon decreasing frequency, the direct current limit (phase approaching zero) is typically reached by ~1 Hz and the resistance was calculated using the final point collected with a frequency of 0.1 Hz. Here, the parallel configurations exhibit resistance values in good agreement: 2307 and 2315 Ω (**A** and **C**, respectively), 1027 and 1020 Ω (**B** and **D**, respectively). The corresponding electronic conductivity per the van der Pauw method is therefore 2.05×10^{-3} S cm^{-1} (see Methods, main text).

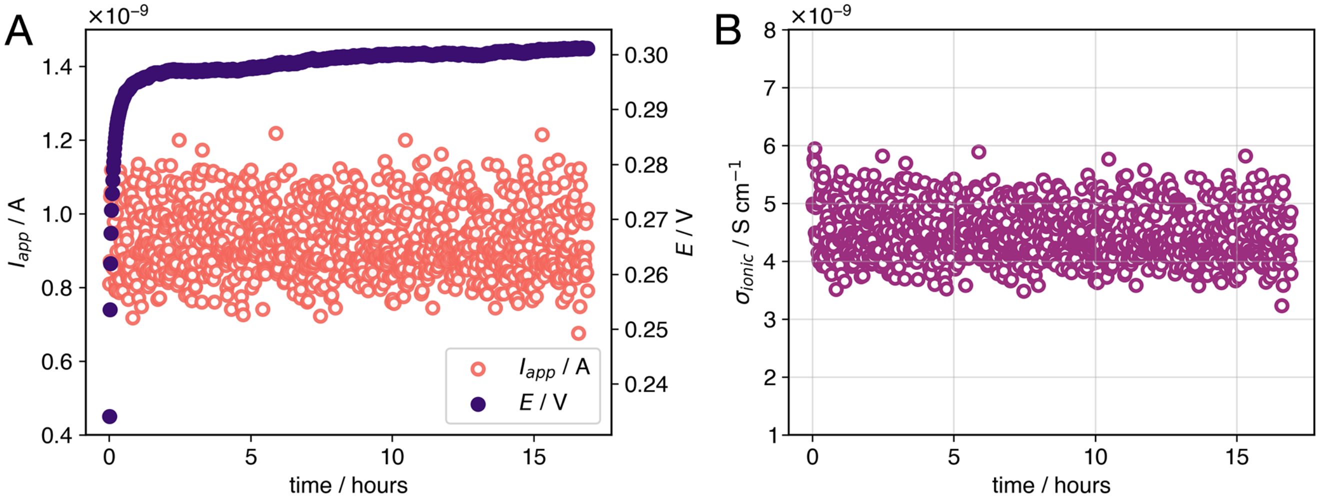

Figure S2. Two-point direct current polarization measurement of a representative Cs_2SnI_6 crystal (thickness = 0.46 mm; contact area = 2.3 mm^2) in a symmetric cell with electron-blocking AgI electrodes (see Figure 1F, main text). The applied current (I_{app}) was 1 nA and the corresponding potential response, E (**A**), was used to calculate the ionic conductivity (**B**, see Methods, main text).

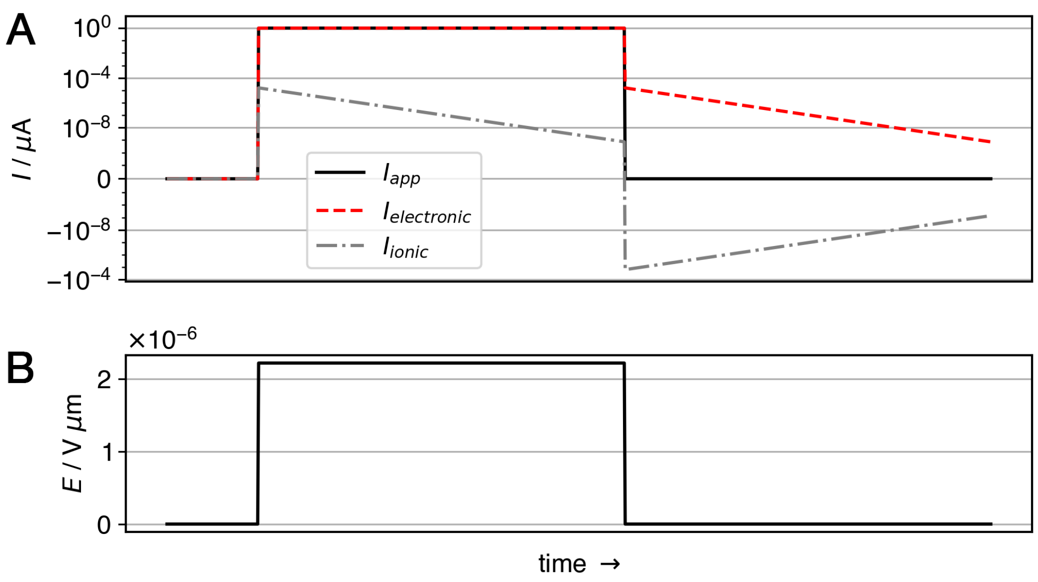

Figure S3. Simulated direct current (**A**) and potential (**B**) profiles corresponding to a hypothetical Cs_2SnI_6 crystal of arbitrary thickness, with electronic and ionic conductivity values of 4.5×10^{-3} and 7.1×10^{-8} S cm^{-1}, respectively (see main text). The simulated deconvolutions into ionic and electronic current components correspond to the polarization response with ideal ion-blocking, electron-conducting electrodes. Due to the large disparity in ionic and electronic conductivity values, Cs_2SnI_6 is a nearly pure electron conductor and therefore exhibits no measurable stoichiometry polarization as observed in some 3D iodide perovskites. See **Simulating transient components of ionic and electronic polarization** (**Supporting Methods**) for a detailed treatment of stoichiometry polarization effects under polarization with selective electrodes. The transient ionic response upon switching the current on is more than four orders of magnitude below the total current and decays to zero at a timescale commensurate with the time constant associated with diffusion (**A**). The switch-off behavior similarly mirrors the small transient response in electronic and ionic current. As a result, the potential follows the current steps in an ideal Ohmic response (**B**).

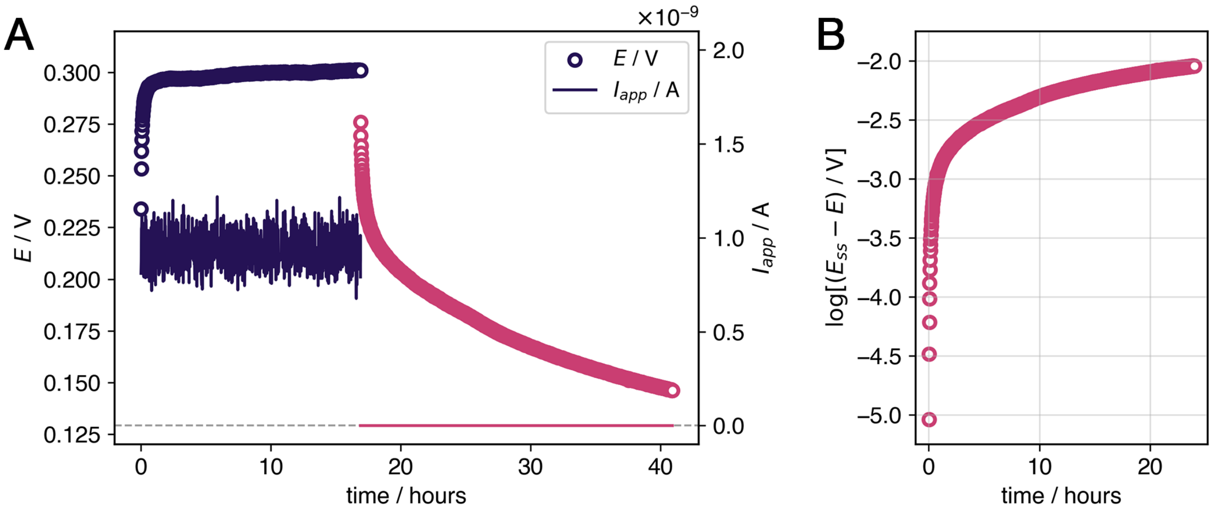

Figure S4. Consecutive direct current polarization and open circuit measurements (**A**) on a representative Cs_2SnI_6 crystal (thickness = 0.46 mm; contact area = 2.3 mm^2) in a symmetric cell with electron-blocking AgI electrodes (see Figure 1F, main text). The observed relaxation in the open circuit potential is analyzed in the long-time limit by a power law transformation (**B**) to obtain the bulk diffusion coefficient (see Figure 1H, main text, and **Supporting Methods**).

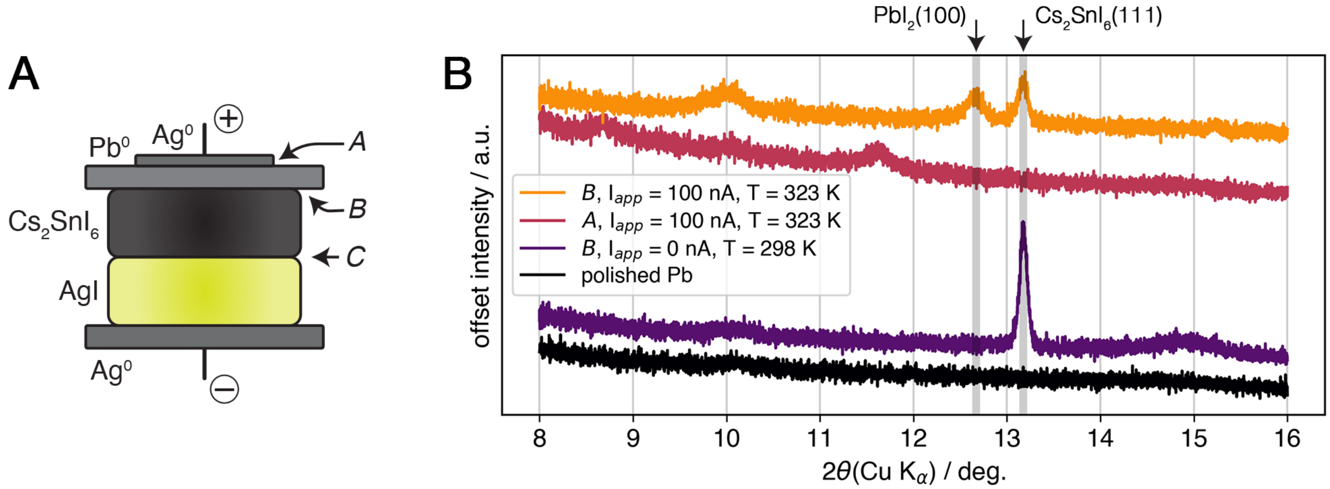

Figure S5. Schematic representation of an asymmetric coulometric reaction cell for determination of the dominant mobile ion in Cs_2SnI_6 (**A**). (**B**) X-ray diffraction patterns from the interfaces of deconstructed coulometric reaction cells (as shown and annotated in A) under various polarization conditions. For experimental details, see **Coulometric reaction cell experiment and characterization** (**Supporting Methods**). Direct current polarization of this reaction cell will drive an interfacial reaction that depends on the dominant mobile ion. Considering the possible mobile ions in Cs_2SnI_6, Cs^+ and Sn^{4+} could precipitate (as CsI, SnI_4, respectively) at interface *C* towards the negative electrode, or I^- could precipitate as PbI_2 at interface *B* towards the positive electrode. Crystalline PbI_2 formation is observed at interface *B* after 7 days of polarization, whereas no PbI_2 formation was observed on the pristine polished Pb electrode or the same interface under no bias; thus, I^- is the dominant mobile ion in Cs_2SnI_6.

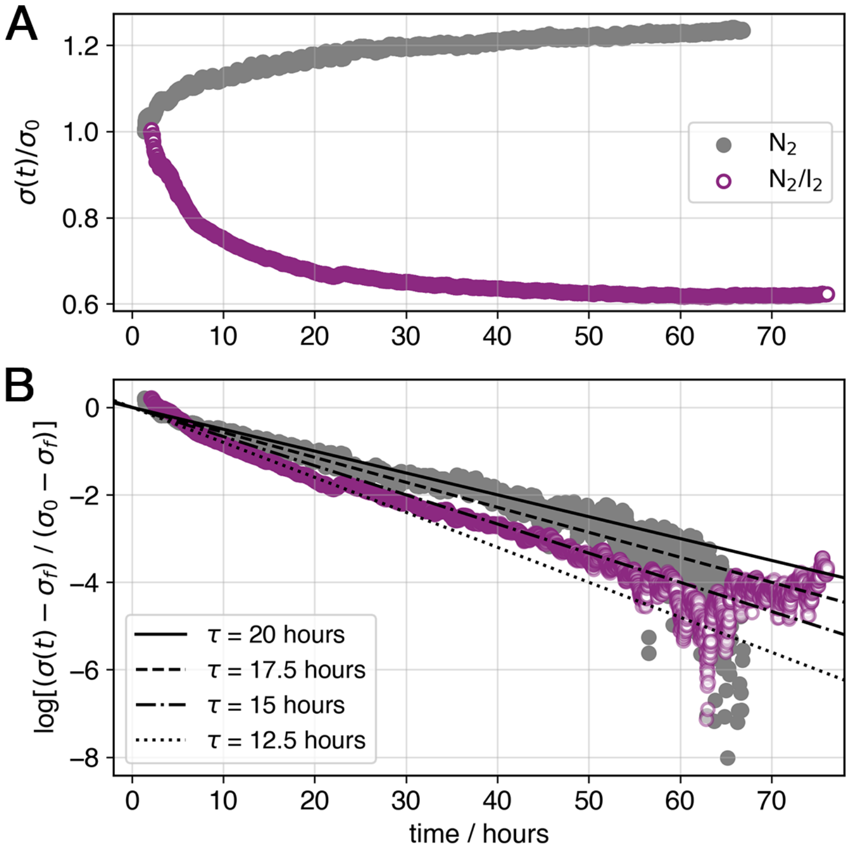

Figure S6. Kinetic analysis of isothermal (~31 °C) electronic conductivity (σ) measurements on a single crystal of Cs_2SnI_6, first under flowing N_2 (1.5 L min^{-1}; denoted N_2) and subsequently after a column of solid I_2 was introduced to the flow for the remainder of the measurement (denoted N_2/I_2), reproduced from Figure 2A (main text). The sequential measurement is shown in Figure 2A and described in detail in the Methods and associated discussion. Here, the data were normalized to the initial conductivity point (σ_0; **A**), demonstrating the mirrored conductivity response under forcing conditions toward opposite directions in the iodine exchange equilibrium (Eq. 2, main text). A power law linearization analogous to that described in the **Supporting Methods** (see **Linearization fitting within the one-dimensional diffusion model formalism**) was also applied to these data (**B**) using σ_0 and the final conductivity point (σ_f); a range of time constants (τ; Eq. 14, main text) associated with diffusion are plotted against the experimental data to guide the eye. The similarity in slope between the two experimental measurements reflects a common time constant associated with diffusion in opposite directions of the exchange equilibrium and supports the assignment of diffusion-limited kinetics. The first two hours of the N_2/I_2 scan were omitted for clarity owing to measurement noise during the gas switch-over upon introduction of I_2, which produces an offset artifact in the power law representation (B).

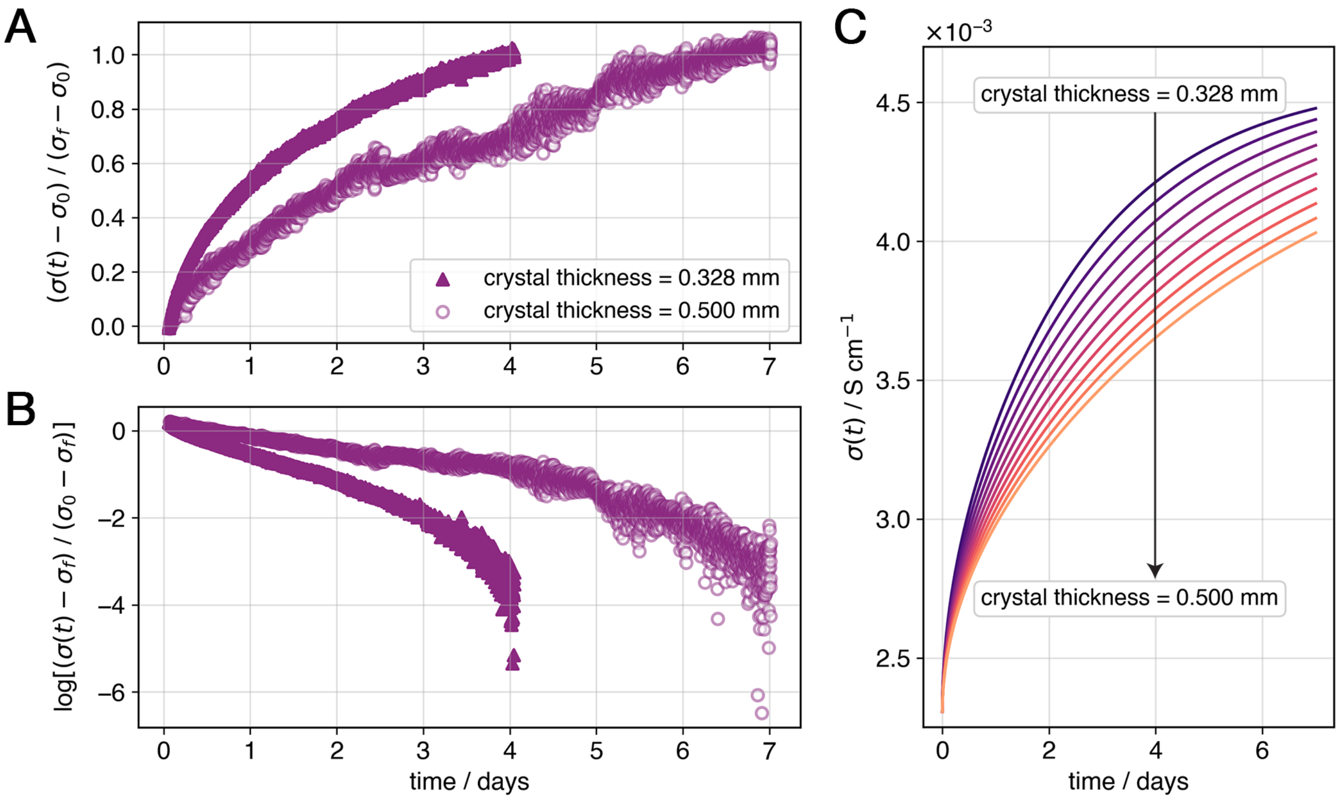

Figure S7. Kinetic analysis of off-gassing electronic conductivity (σ) measurements on a single crystal of Cs_2SnI_6 before (crystal thickness = 0.500 mm) and after (crystal thickness = 0.328 mm) high-$p(I_2)$ treatment and cleaving to expose a fresh surface, reproduced from Figure 2D (main text). The comparison here highlights measurements collected at a similar temperature of T = 40-41°C; the full measurement is shown in Figure 2D and described in detail in the Methods and associated discussion. Here, the data were scaled by the initial and final conductivity points (σ_0 and σ_f, respectively; **A**). A power law linearization analogous to that described in the **Supporting Methods** (see **Linearization fitting within the one-dimensional diffusion model formalism**) was also applied to these data (**B**). Both representations (A, B) reflect a change in time constant (Eq. 14, main text) consistent with the reduced thickness, provided the diffusion coefficient is constant. Indeed, simulation of off-gassing traces (Eq. 3, main text) with thickness values between 0.328 and 0.500 mm (with all other parameters held constant: $D = 5 \times 10^{-10}$ cm^2 s^{-1}; $c_b^* = 2 \times 10^{15}$ cm^{-3}; $c_s^* = 4 \times 10^{15}$ cm^{-3}; $\mu_e = 7.17$ cm^2 V^{-1} s^{-1}) reflects a similar modulation of the conductivity response (**C**).

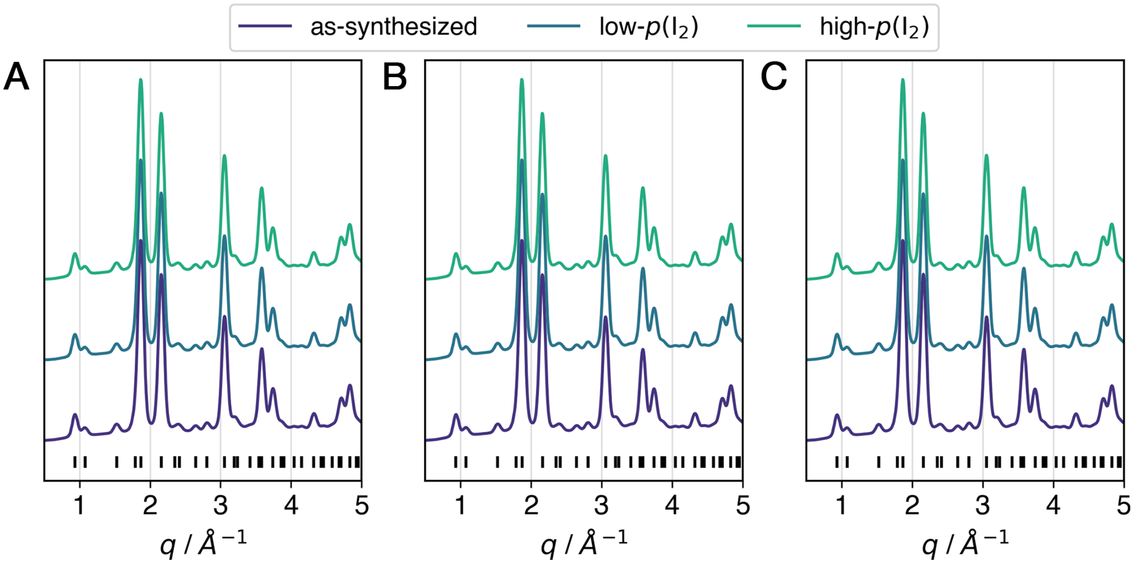

Figure S8. Powder X-ray diffraction patterns collected from polycrystalline Cs_2SnI_6 powders from three synthetic batches (**A**-**C**) in three states representative of the variable-$p(I_2)$ treatments highlighted in the main text (see Methods). The observed Bragg reflections are all indexed to Cs_2SnI_6 (*Fm–3m*), where the positions of allowed reflections (in q) are included for reference (black vertical lines).

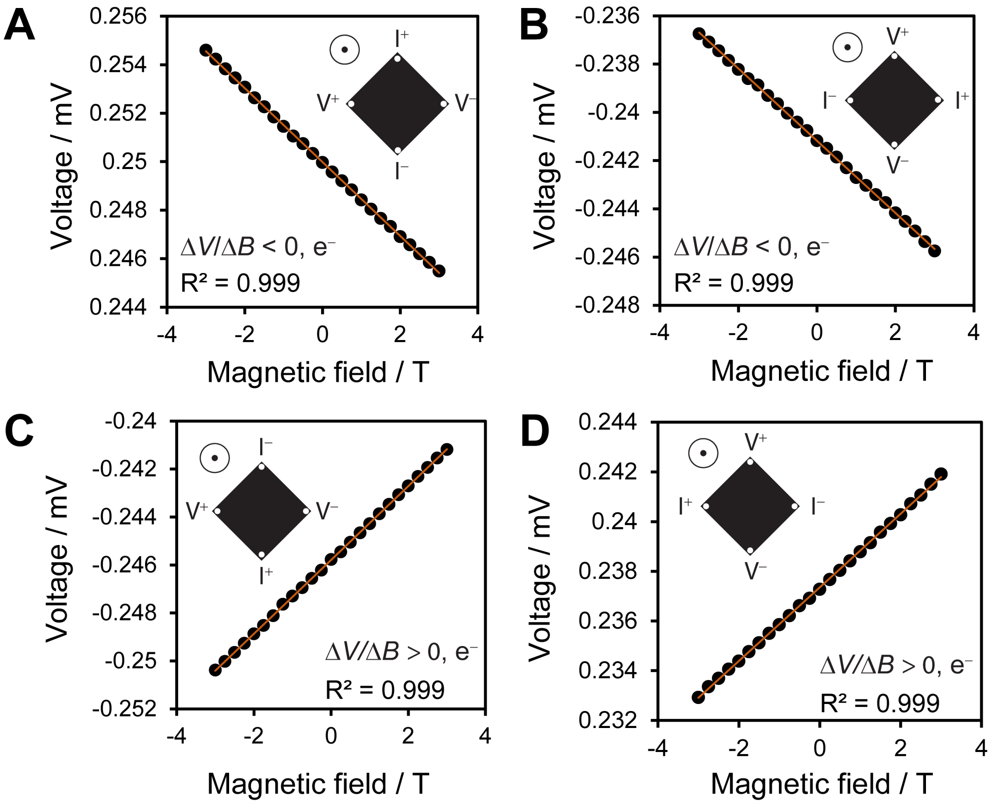

Figure S9. Variable-field Hall effect measurements on the low-*p*(I_2) Cs_2SnI_6 crystal (see Figure 3 and accompanying discussion, main text) at 300 K. The voltage is shown after the zero-field drift correction—for measurements between –3 T and 3 T with a current of 0.1 μA—in each of the four Hall configurations (**A-D**, see inset and **Figure S11**). The expected sign of the Hall voltage, or the slope *ΔV/ΔB*, is shown in the case that electrons (e^-) are the majority carriers.

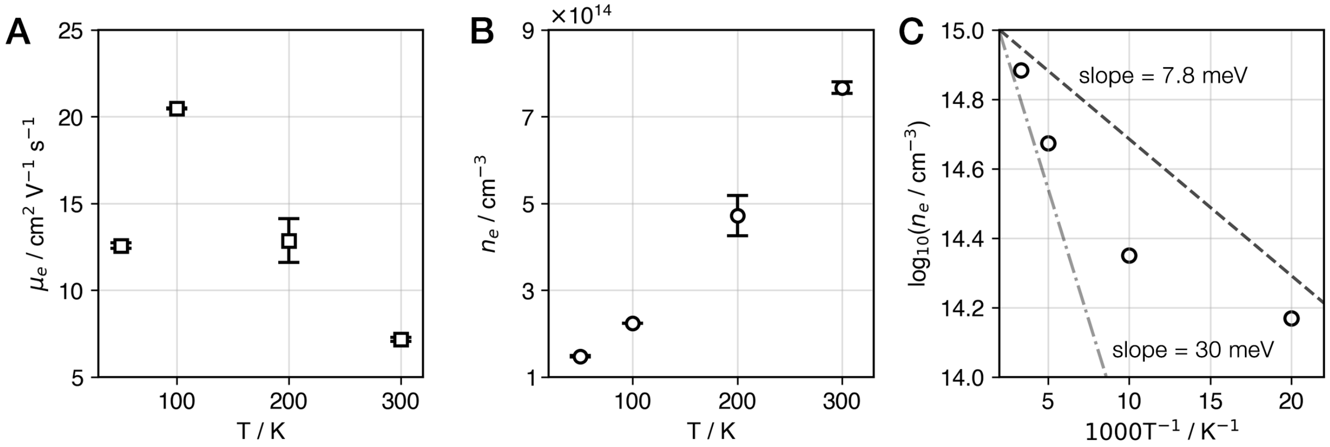

Figure S10. Electron mobility (μ_e, **A**) and concentration (n_e, **B**) quantified by analysis of Hall effect measurements on the low-$p(I_2)$ crystal; error bars represent one standard deviation. (**C**) Logarithmic plot of n_e, measured on the low-$p(I_2)$ crystals, and slopes corresponding to the bounds of the donor level quantified by fitting the temperature-dependent conductivity measurements in the freeze-out region (see Figure 3 and discussion, main text).

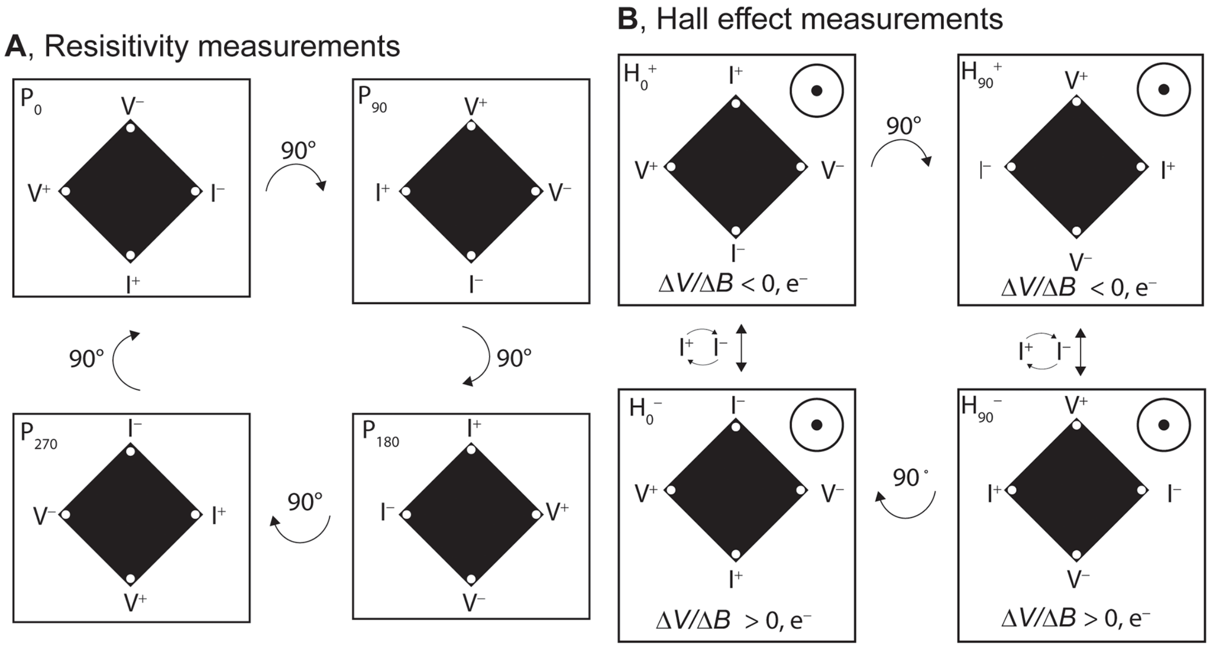

Figure S11. Configuration of the voltage channels (V^+, V^-) and the electrical source channels (I^+, I^-) for resistivity measurements (**A**) and Hall effect measurements (**B**). The direction of the applied magnetic field is indicated in the upper right-hand corner for the Hall configurations and the expected sign of the Hall voltage, or the slope $\Delta V/\Delta B$, is given in the case that electrons (e^-) are the majority carriers.

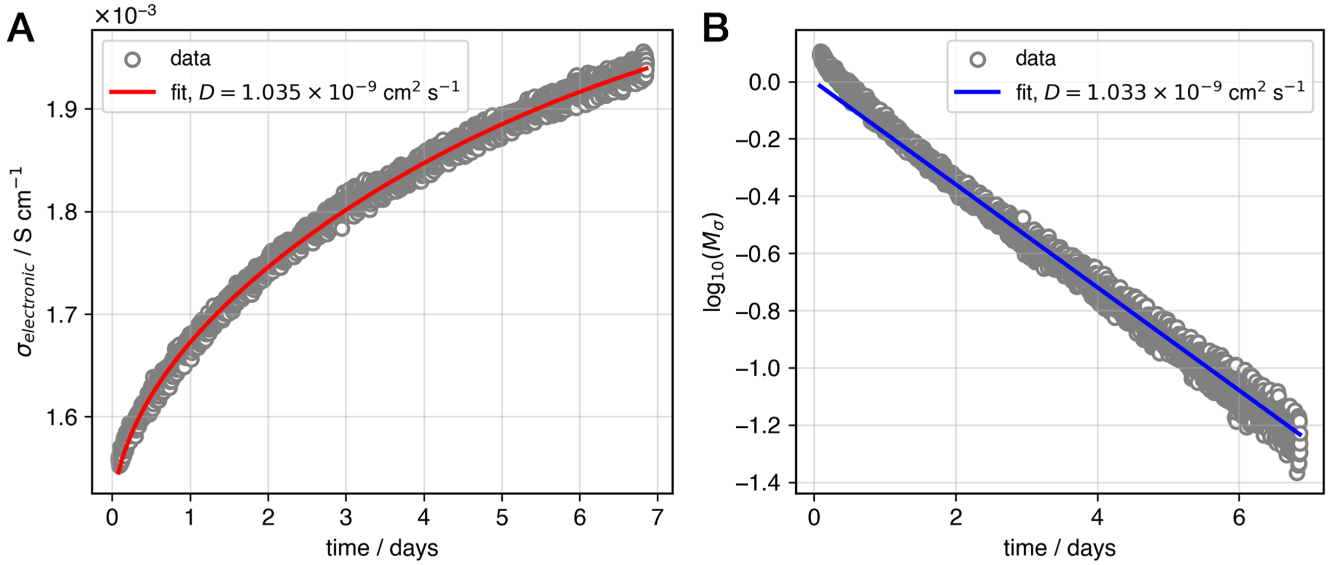

Figure S12. Representative fits to an off-gassing conductivity measurement ($\sigma_{electronic}$ reproduced from Figure 4A, main text; 53 °C trace), highlighting the agreement between diffusion coefficient (*D*) determination using the complementary full-model fitting (**A**; see Methods, main text) and linearization fitting (**B**; $M_\sigma = (\sigma_{electronic} - e|z|\mu_e c_b^*)/[e|z|\mu_e(c_s^* - c_b^*)]$; see **Supporting Methods**) approaches.

Supporting Tables I

Table S1. Measured temperature and model fit parameters from the off-gassing traces shown in Figure 2D (main text). The first off-gassing conductivity measurements, **1**, in the left panel of Figure 2D were modeled with constant values of L = crystal thickness = 0.500 mm and μ_e = electron mobility = 7.17 cm^2 V^{-1} s^{-1}. The second off-gassing conductivity measurements after exposure to I_2(s, sat.), **2**, in the right panel of Figure 2D were modeled with constant values of L = 0.328 mm and μ_e = 7.17 cm^2 V^{-1} s^{-1}.

Index	Temperature / K	Fit D / cm^2 s^{-1}	Fit c_s^* / cm^{-3}	Fit c_b^* / cm^{-3}
1	303.4(3)	1.0×10^{-9}	3.05×10^{15}	1.79×10^{15}
1	312.8(4)	4.2×10^{-10}	4.18×10^{15}	3.01×10^{15}
1	323.5(3)	3.6×10^{-10}	5.92×10^{15}	3.77×10^{15}
2	314.2(4)	6.7×10^{-10}	3.45×10^{15}	1.79×10^{15}
2	326.6(5)	2.8×10^{-10}	5.72×10^{15}	3.25×10^{15}

Table S2. Hall voltages determined from Hall effect measurements on the low-$p(I_2)$ Cs_2SnI_6 crystal (see Figure 3 and accompanying discussion, main text). H_0^+, H_{90}^+, H_0^-, H_{90}^- are the four Hall configurations (see **Figure S11**), V = voltage, B = magnetic field.

Temperature / K	H_0^+ / µV K^{-1}	H_0^- / µV K^{-1}	H_{90}^+ / µV K^{-1}	H_{90}^- / µV K^{-1}	$(\Delta V/\Delta B)^{average}$ / µV K^{-1}
300	1.52	1.54	1.50	1.48	1.51(3)
200	2.28	--	2.62	--	2.4(2)
100	5.16	--	5.17	--	5.17(1)
50	7.92	--	7.78	--	7.85(9)

Table S3. Measured temperature and model fit parameters from the off-gassing traces shown in Figure 4A (main text). The off-gassing conductivity measurements were modeled with constant values of L = 0.7 mm and μ_e = 7.17 cm^2 V^{-1} s^{-1}.

Temperature / K	Fit D / cm^2 s^{-1}	Fit c_s^* / cm^{-3}	Fit c_b^* / cm^{-3}
305.6(5)	2.3×10^{-9}	1.03×10^{15}	6.52×10^{14}
314.7(5)	9.7×10^{-10}	1.39×10^{15}	1.03×10^{15}
325.8(5)	1.0×10^{-9}	1.81×10^{15}	1.30×10^{15}
336.7(7)	1.2×10^{-9}	2.22×10^{15}	1.69×10^{15}
348.3(8)	2.6×10^{-9}	2.49×10^{15}	2.10×10^{15}

Supporting Discussion

S. D. 1. Alternative representations of the exchange equilibrium and their implications

In this work, we proposed and investigated the iodine exchange equilibrium in the context of the dominant mobile ion and defect in Cs_2SnI_6 (see main text, **Eq. 2** and associated discussion):

$$nil \leftrightarrow 2V_I^{\bullet} + I_{2\,(g)} + 2e'$$

Here, we confirmed experimentally that I^- is the dominant mobile ion (see main text, **Structure and transport properties of Cs_2SnI_6** and **Figure S5**). Further, beyond the overwhelming precedent from prior investigations of related 3D iodide perovskites[3, 5, 6, 8, 9] that the defect associated with I^- migration (the dominant defect) is the iodine vacancy (V_I), V_I formation is energetically favored relative to *every* other intrinsic point defect in Cs_2SnI_6.[10] We can therefore analyze the iodine exchange equilibrium with this rationalized assumption that V_I is the mediating point defect (see above). More generally, it bears mentioning that the exchange equilibrium can be represented in myriad reactions involving either a single point defect or plausible defect pairs; such considerations have been applied in rigorous defect chemical analysis of oxides as a function of external $p(O_2)$.[11] A similar analysis leads to representation of the iodine exchange equilibrium with charge-neutral iodine interstitials (I_i^x) as the dominant defect:

$$2I_i^x \leftrightarrow I_{2\,(g)}$$

The interstitial defect can be ionized under certain conditions and the mononegative interstitial would produce an analogous self-doping effect, albeit with holes (*p*-type). This reaction was discussed in a report investigating I_2-doping in polycrystalline $(CH_3NH_3)PbI_3$.[12] Specific to $(CH_3NH_3)PbI_3$, contemplating an iodine interstitial is justified considering[12, 13]: (*i*) the similar formation enthalpy values for iodine vacancies and interstitials; (*ii*) the iodine interstitial is expected to manifest as a shallow electron acceptor; (*iii*) $(CH_3NH_3)PbI_3$ is most often *p*-type or near the intrinsic regime. Nonetheless, prior elucidation of the dominant defect as V_I in $(CH_3NH_3)PbI_3$[5] led the authors to also analyze iodine-induced doping involving iodine vacancies (above and main text, **Eq. 2**).

The defect thermodynamics of Cs_2SnI_6 suggest that participation of iodine interstitial defects is significantly less likely than in $(CH_3NH_3)PbI_3$. First, calculated formation enthalpy values for iodine interstitials (2.54–3.0 eV) are markedly higher than for iodine vacancies (0.28–0.74 eV).[10] Second, these calculations also suggest that the iodine interstitial is a deep acceptor and may therefore be charge-neutral at the relevant Fermi level; a neutral interstitial defect may exchange with the atmosphere (above) but would not produce the self-doping effect we investigated. Finally, a hypothetically dominant iodine interstitial defect would suggest that an *n-i-p* transition should be observed with increasing $p(I_2)$.[11] It is theoretically possible that this transition occurs outside of an accessible stability window, however no experimental evidence (to our knowledge) exists of *i* or *p*-type Cs_2SnI_6 across a range of synthetic conditions. Indeed, the iodine vacancy formation enthalpy is lower than that of the interstitial defect (by 1.8 eV or more) along *all* boundaries of the thermodynamic stability of Cs_2SnI_6.[10] Thus, we can assume

that the iodine vacancy is the dominant defect mediating iodine transport and exchange, and we do not consider further the implications of interstitial-based iodine exchange reactions.

S. D. 2. Internal defect chemistry of Cs_2SnI_6: compensation and the total vacancy concentration

A defect reaction classified as *external* is observed and discussed in detail in the main text, namely iodine/iodide exchange. The exchange of I_2 gas in the atmosphere and iodide anions in a Cs_2SnI_6 crystal occurs through the solid-gas interface after the crystal has been removed from the mother liquor of the crystallization. Of course, there also exist *internal* point defects in the as-grown crystal that form before the effects of the external iodine exchange reaction become relevant in an open system. The possible internal point defects in Cs_2SnI_6 and calculated energies are presented elsewhere,[10] and we note that Schottky-type vacancy disorder (involving Cs and I) appears to be most prevalent upon comparison of calculated formation enthalpies.[10, 14] The predominant internal disorder is relevant to the broader discussion—including the iodine/iodide exchange reaction—owing to the role of compensating defects and the fraction of iodine vacancies that exist prior to iodine off-gassing, which affect the external exchange equilibrium.

To model the internal defect chemistry with the electronic property measurements undertaken within our study, we constructed a system of three non-linear equilibrium relations and an electroneutrality condition. First, the excitation of free charge carriers (e' and $h^\bullet$) is expressed in terms of the fundamental bandgap (E_g):

$$[e'][h^\bullet] = e^{-\frac{E_g}{k_b T}}$$

Concentrations are denoted by square brackets. Here, as noted above, we assume that a low-formation enthalpy Schottky reaction dominates the internal disorder, consistent with the abundance of iodine vacancies that we observe. Our choice to model cesium vacancies in this regard (to charge-balance iodine vacancies in the Schottky reaction) can be considered a placeholder for an arbitrary compensating defect. Indeed, the conclusions are not affected by this choice and future studies may illuminate the chemical identity and energy levels of the compensating defects in Cs_2SnI_6. The Schottky disorder during crystallization is represented in defect notation as,

$$nil \leftrightarrow V_I^\bullet + V_{Cs}'$$

where *nil* is the perfect crystal and $V_I^\bullet$ and V_{Cs}' are charged (+1 / –1, respectively) iodine and cesium vacancies, respectively. The relevant equilibrium expression for the formation of such Schottky defects depends on the concentration (or activity, *a*) of CsI in the crystallization solution and the free energy of formation (ΔG_f):

$$a[V_{Cs}'][V_I^\bullet] = e^{-\frac{\Delta G_f}{k_b T}}$$

Here and above the vacancies are expressed in an ionized state; however, the ionization energy (ΔE) was determined for the iodine vacancy donor through analysis of electronic property measurements (see main text, Figure 3, Eq. 5, and associated discussion). Main text Eq. 5 can be re-formulated in this context to describe the ionization,

$$\frac{[V_I^{\bullet}][e']}{[V_I^x]} = e^{-\frac{\Delta E}{k_b T}}$$

where V_I^x is a neutral iodine vacancy and e' is a free conduction-band electron after ionization from the donor level. The ionized vacancy concentration $[V_I^{\bullet}]$ is estimated from the ionic conductivity (see main text Figure 2 and associated discussion), given the conductivity is vacancy-mediated. Finally, an electroneutrality condition,

$$[V_{Cs}'] + [e'] = [V_I^{\bullet}] + [h^{\bullet}]$$

including electronic charges and ionized intrinsic defects is defined to ensure overall charge balance.

The system was solved and analytical solutions were evaluated using custom python code based on the SymPy package.[15] Relevant values for physical constants and quantities used to numerically evaluate the solutions are given in **Table S4**. We evaluated the solutions with two values for ΔE (**Table S5**), in line with our findings shown in Figure 3 (main text), **Figure S10**, and associated discussions related to the quantification of the donor ionization energy. Both solutions point to the abundance of iodine vacancies and compensating cesium vacancies. Again, the choice to model cesium vacancies as the compensating defect is rather arbitrary and based solely on the likely prevalence of Schottky disorder and a comparison of the calculated formation energies of point defects in Cs_2SnI_6.[10] The iodine vacancy concentration is constrained by the observed bulk diffusion coefficient and ionic conductivity, and the result is a large estimated vacancy concentration of ~1.5×10^{19} cm^{-3}. The iodine vacancy donor ionization energy (7.8-30 meV) further suggests that nearly all vacancies are ionized, and the crystal therefore must be compensated by a negatively charged defect at a concentration far above that of the free electrons (7.67 $\times 10^{14}$ cm^{-3}). Thus, we conclude that as-synthesized Cs_2SnI_6 likely exhibits intrinsic compensation, and the electronic consequences are discussed in the following section (**S.D.3**). Furthermore, the high intrinsic vacancy concentration motivates our description of additional vacancies produced through the external off-gassing reaction as *excess* vacancies, given the disparity in concentrations (~10^{19} cm^{-3} intrinsic, ~10^{15} external through the iodine exchange equilibrium). It is worth noting that the quantities produced by evaluating solutions from this model rely on values from the ionic property measurements; a limiting assumption here is that the diffusion coefficient corresponds to iodide transport exclusively through iodine vacancies. If this is not the case (*e.g.*, iodide ions diffuse through interstitial sites, or other pathways), the total vacancy concentration is likely overestimated. Thus, we are careful not to over-interpret the precise level of compensation in the crystal.

S. D. 3. The effect of compensation on electronic properties dominated by an electron donor

Significant compensation from intrinsic point defects results in a complex dynamic between donors, acceptors, and free electronic charge carriers in a semiconductor. Here, we refer to compensation as a state where a charge-balancing point defect *compensates* the primary dopant or charged defect, or where the difference in donor and acceptor densities is sufficiently small.[13] Others have noted the prevalence of compensation in halide perovskites, particularly as a result of facile intrinsic point defect formation.[8, 13] In the previous section (**S.D.2**), the evaluation of our internal defect chemical model indicated a high likelihood of compensation in Cs_2SnI_6. The electronic properties discussed in the main text are consistent with this conclusion; however, before discussing these aspects, it is worthwhile—for context—to consider the ideal behavior of an n-type semiconductor dominated by an electron donor.

The theory for a single monovalent donor species (*e.g.*, V_I) is presented in detail by Blakemore,[16] and we summarize the salient points for the sake of discussion. This is the simple picture represented schematically in Figure 3E of the main text, with a single donor level proximal to the conduction band minimum (E_C) and without consideration of the presence of acceptors. In the extrinsic region (intermediate temperatures between dopant freeze-out and temperatures sufficient for intrinsic carrier generation) and at higher temperatures (including intrinsic carrier generation), the Fermi level is between the donor energy (E_d) and the intrinsic Fermi level (approximately half-way between E_C and the valence band maximum, depending more precisely on the relative effective density of states in the conduction band and valence band). As the temperature decreases to be comparable to the ionization energy ($\Delta E = E_C - E_d$) and below, free electron generation is dominated by the donor and the Fermi level increases toward E_C. The temperature dependence of the conduction-band electron concentration (n) in this region scales with $\Delta E/2$:

$$\log(n) \propto -\frac{\Delta E}{2k_B T}$$

The presence of compensating acceptors changes the fundamental balance of charge and—among other considerations—changes the temperature dependence of n. When the concentration of acceptors is sufficiently small (much less than n), the above expression still applies. Alternatively, when n is less than the concentration of acceptors, the corresponding relation scales with ΔE:

$$\log(n) \propto -\frac{\Delta E}{k_B T}$$

Thus, given that we are unable to determine the level of compensation directly, our estimation of the activation energy from electronic conductivity measurements (see main text Figure 3D and associated discussion) and Hall effect measurements of n (**Figure S10**) can only place bounds on the value of ΔE (7.8-30 meV).

Finally, we note that compensation may be related to the degenerate semiconducting behavior observed in single crystals of Cs_2SnI_6 near room temperature (see main text Figure 3B and associated discussion). Compensation effectively reduces the apparent majority carrier concentration despite a significant degree of ionic and electronic disorder. In this case, ionic conductivity measurements and defect modeling (see **S.D.2**) suggest that the ionized donor and compensating acceptor concentrations are more than three orders of magnitude higher than the free electron concentration (**Table S4** and **S5**). So, whereas a semiconductor dominated by an extrinsic donor without compensation (or with a donor ionization energy above 30 meV) should exhibit ideal non-degenerate behavior near room temperature at $n \cong 10^{15}$ cm^{-3}, the Fermi level may be closer to E_C—giving rise to degenerate semiconducting behavior—in a Cs_2SnI_6 crystal with $n \cong 10^{15}$ cm^{-3} that exhibits heavy compensation. Furthermore, as discussed in the main text, the thermal ionization energy of the dominant donor/acceptor in heavily doped semiconductors decreases with increased doping due to charge screening and Coulomb interactions,[17-19] consistent with our observations for Cs_2SnI_6 under variable $p(I_2)$ (see Figure 3D, main text, and associated discussion). Electrostatic models accounting for compensating defects also predict a depression in the ionization energy with a greater degree of compensation (in addition to the reduction due to increased donors).[19] The metal-like charge transport characteristic of degenerate doping in select temperature ranges is often attributed to related many-body interactions when an ionized defect reaches concentrations of ~10^{18} cm^{-3} (with a precise threshold depending on the composition and electronic structure). At these concentrations, defect-defect and defect-carrier interactions can produce bandgap narrowing, tailing, and the formation of a dispersive donor band with states at various energies (rather than a "flat" defect level at one energy, E_d), where the donor population can approach and ultimately extend into the conduction band. We refer the interested reader to the aforementioned texts[16, 17] and review articles[18, 20] for a further discussion of the impacts of degenerate doping in semiconductors.

S. D. 4. Comparing the thermodynamics of oxygen and halogen exchange

We presented here the first characterization of the thermodynamics of a halogen/halide exchange defect reaction with an appreciation for the abundance of studies on the analogous oxygen/oxide exchange reaction (Eq. 1, main text). Indeed, comparing the thermodynamic quantities we estimate here with those from ceramics yield insight into composition-dependent defect chemistry and the nature of external defect reactions mediated through the solid-gas interface. These aspects are discussed in detail in the main text (see *Quantifying the spontaneity of the off-gassing reaction* and *Discussion*). One salient difference between the two classes of materials is the extent of non-stoichiometry (denoted δ) that external exchange induces. In oxides, δ (in, for instance, $ABO_{3-\delta}$) can surpass 10% without triggering a phase change or decomposition. The thermodynamics of oxygen exchange are not constant across such a wide range of stoichiometry, and thus the thermodynamic quantities depend on δ. In addition, configurational entropy begins to play a significant role in the solid above small values of δ. Experimentally determined values of the partial molar enthalpy and entropy are nonetheless comparable between these reactions and reveal key trends in the thermodynamics. **Table S6** compiles representative quantities from the literature for select oxides, specifically fluorites and perovskites, as a comparison to Cs_2SnI_6. Owing to the reducibility of the metal ions in oxides, electrons produced in these reactions are often localized and Eq. 1 (main text) is

considered a formal reduction. Values reported as partial molar quantities of oxidation (*e.g.*, for $Ba_{0.5}Sr_{0.5}Co_{0.8}Fe_{0.2}O_3$[21]) were converted to align with the convention used here and elsewhere, where quantities are reported for the forward reaction (Eq. 1, 2, main text).

The large disparity in enthalpy and agreement in entropy are most striking when comparing the oxides and Cs_2SnI_6 (**Table S6**). The enthalpy of reaction varies with composition, structure, and δ for the oxides, but tends to be higher than 1.5 eV; alternatively, the enthalpy associated with I_2 loss in Cs_2SnI_6 is only 0.38 eV. This is a significant difference—that we credit for the observation of room-temperature off-gassing (see main text)—but generally agrees with the difference in point defect formation energies between oxide and halide perovskites. Indeed, these differences have been attributed to the higher average charge and lattice energy in oxide crystals.[8, 13] So, while the enthalpy associated with off-gassing may vary with composition across halide perovskites (although the group-14 *B*-site metals such as Pb, Sn, and Ge exhibit similar metal-halogen bond enthalpies; see main text), there is good reason to expect that the magnitude is systematically lower in halides (compared to oxides). Furthermore, the entropy of reaction is consistent across all materials we compare here (**Table S6**), reflecting the favorability of gas formation in the forward reaction of the equilibrium. In contrast to the oxides, we expect that the configurational enthalpy contribution is minor for the halide perovskites, which exhibit a relatively small accessible range of halide non-stoichiometry. Both components of the free energy therefore suggest that spontaneous room-temperature halogen off-gassing is general to halide perovskites, and the low bandgap and tunable electronic structure in these materials call for consideration of the role of this external defect equilibrium on electronic and ionic transport properties.

Supporting Tables II

Table S4. Physical constants and quantities used in the internal defect chemical modeling of Cs_2SnI_6.

Quantity	Numerical value	Reference
Density	4.84 g cm^{-3}	—
Formula weight	1145.4 g mol^{-1}	—
Electron effective mass	$0.48m_0$	14
Hole effective mass	$1.32m_0$	14
Fundamental bandgap	1.3 eV	14, 22
Electron concentration (300 K)	7.67×10^{14} cm^{-3}	this work
Diffusion coefficient, I^-	7.65×10^{-10} cm^2 s^{-1}	this work
Ionic conductivity	7.1×10^{-8} S cm^{-1}	this work
Electronic conductivity	4.5×10^{-3} S cm^{-1}	this work
Activity of CsI in solution	0.2 mol L^{-1}	this work

Table S5. Numerical evaluation of the internal defect chemical model solutions, with two values for the iodine vacancy donor ionization energy (ΔE).

Quantity	Numerical solution for ΔE = 7.8 meV	Numerical solution for ΔE = 30 meV
Charge-neutral iodine vacancy concentration, $[V_I^x]$	1.85×10^{15} cm^{-3}	4.37×10^{15} cm^{-3}
Hole concentration, $[h^\bullet]$	5.99×10^{1} cm^{-3}	5.99×10^{1} cm^{-3}
Ionized (–1 charge) cesium vacancy concentration, $[V_{Cs}{}']$	1.49×10^{19} cm^{-3}	1.49×10^{19} cm^{-3}
Free energy of formation of the $[V_{Cs}{}'] - [V_I{}^\bullet]$ Schottky defect pair, ΔG_f	0.372 eV	0.372 eV

Table S6. Partial molar enthalpy (ΔH^r) and entropy (ΔS^r) of reaction (per mole of X_2, X = O, I) of reaction for the generation of X_2 and vacancies in the solid crystal, for various oxides and the iodide double perovskite Cs_2SnI_6.

Nominal composition (form)	ΔH^r / eV	ΔS^r / eV K^{-1}	Reference
Cs_2SnI_6 (single crystal)	0.38	1.3×10^{-3}	this work
CeO_2 (polycrystalline powder)	4.98	—	23
$Sm_{0.1}Ce_{0.9}O_{1.95}$ (polycrystalline powder)	4.15	—	23
$Sm_{0.2}Ce_{0.8}O_{1.9}$ (polycrystalline powder)	3.99	—	23
$Gd_{0.1}Ce_{0.9}O_{1.95}$ (polycrystalline powder)	4.54	—	23
$Gd_{0.2}Ce_{0.8}O_{1.9}$ (polycrystalline powder)	3.99	—	23
$Sm_{0.15}Ce_{0.85}O_{1.925}$ (epitaxial film)	4.16-4.19	$1.10\text{-}1.19 \times 10^{-3}$	24
$Ba_{0.5}Sr_{0.5}Co_{0.8}Fe_{0.2}O_3$ (polycrystalline powder)	1.8-2.5	$1.5\text{-}2.0 \times 10^{-3}$	21
$La_{0.6}Sr_{0.4}Co_{0.2}Fe_{0.8}O_3$ (polycrystalline powder)	1.7-2.2	$1.5\text{-}1.8 \times 10^{-3}$	21
$La_{0.6}Sr_{0.4}MnO_3$ (polycrystalline powder)	0.4-3.2	$0.3\text{-}1.4 \times 10^{-3}$	25

Supporting References

(1) Ashiotis, G.; Deschildre, A.; Nawaz, Z.; Wright, J. P.; Karkoulis, D.; Picca, F. E.; Kieffer, J. The fast azimuthal integration Python library: pyFAI. *J. Appl. Crystallogr.* **2015**, *48* (2), 510-519.

(2) Maier, J. Kinetics and Irreversible Thermodynamics. In *Physical Chemistry of Ionic Materials*, John Wiley & Sons, Ltd, 2004; pp 268-398.

(3) Yang, T.-Y.; Gregori, G.; Pellet, N.; Grätzel, M.; Maier, J. The Significance of Ion Conduction in a Hybrid Organic–Inorganic Lead-Iodide-Based Perovskite Photosensitizer. *Angew. Chem. Int. Ed.* **2015**, *54* (27), 7905-7910.

(4) Maier, J. Solid State Electrochemistry: Measurement Techniques and Applications. In *Physical Chemistry of Ionic Materials*, John Wiley & Sons, Ltd, 2004; pp 399-499.

(5) Senocrate, A.; Moudrakovski, I.; Kim, G. Y.; Yang, T.-Y.; Gregori, G.; Grätzel, M.; Maier, J. The Nature of Ion Conduction in Methylammonium Lead Iodide: A Multimethod Approach. *Angew. Chem. Int. Ed.* **2017**, *56* (27), 7755-7759.

(6) Matheu, R.; Vigil, J. A.; Crace, E. J.; Karunadasa, H. I. The halogen chemistry of halide perovskites. *Trends Chem.* **2022**, *4* (3), 206-219.

(7) Mizusaki, J.; Arai, K.; Fueki, K. Ionic conduction of the perovskite-type halides. *Solid State Ion.* **1983**, *11* (3), 203-211.

(8) Walsh, A.; Scanlon, D. O.; Chen, S.; Gong, X. G.; Wei, S.-H. Self-Regulation Mechanism for Charged Point Defects in Hybrid Halide Perovskites. *Angew. Chem. Int. Ed.* **2015**, *54* (6), 1791-1794.

(9) Senocrate, A.; Maier, J. Solid-State Ionics of Hybrid Halide Perovskites. *J. Am. Chem. Soc.* **2019**, *141* (21), 8382-8396.

(10) Xiao, Z.; Zhou, Y.; Hosono, H.; Kamiya, T. Intrinsic defects in a photovoltaic perovskite variant Cs2SnI6. *Phys. Chem. Chem. Phys.* **2015**, *17* (29), 18900-18903.

(11) Maier, J. Equilibrium Thermodynamics of the Real Solid. In *Physical Chemistry of Ionic Materials*, 2004; pp 108-267.

(12) Zohar, A.; Levine, I.; Gupta, S.; Davidson, O.; Azulay, D.; Millo, O.; Balberg, I.; Hodes, G.; Cahen, D. What Is the Mechanism of MAPbI3 p-Doping by I2? Insights from Optoelectronic Properties. *ACS Energy Lett.* **2017**, *2* (10), 2408-2414.

(13) Euvrard, J.; Yan, Y.; Mitzi, D. B. Electrical doping in halide perovskites. *Nat. Rev. Mater.* **2021**, *6* (6), 531-549.

(14) Maughan, A. E.; Ganose, A. M.; Bordelon, M. M.; Miller, E. M.; Scanlon, D. O.; Neilson, J. R. Defect Tolerance to Intolerance in the Vacancy-Ordered Double Perovskite Semiconductors Cs2SnI6 and Cs2TeI6. *J. Am. Chem. Soc.* **2016**, *138* (27), 8453-8464.

(15) Meurer, A.; Smith, C. P.; Paprocki, M.; Čertík, O.; Kirpichev, S. B.; Rocklin, M.; Kumar, A.; Ivanov, S.; Moore, J. K.; Singh, S.; et al. SymPy: symbolic computing in Python. *PeerJ Comput. Sci.* **2017**, *3*, e103.

(16) Blakemore, J. S. Semiconductors Dominated by Impurity Levels. In *Semiconductor Statistics*, Pergamon, 1962; pp 117-176.

(17) Pierret, R. F. Degenerate Semiconductor Considerations. In *Advanced Semiconductor Fundamentals*, Pearson Education, Inc., pp 127-128.

(18) Van Overstraeten, R. J.; Mertens, R. P. Heavy doping effects in silicon. *Solid State Electron.* **1987**, *30* (11), 1077-1087.

(19) Poklonskii, N. A.; Syaglo, A. I.; Biskupski, G. A model of how the thermal ionization energy of impurities in semiconductors depends on their concentration and compensation. *Semiconductors* **1999**, *33* (4), 402-406.

(20) Freysoldt, C.; Grabowski, B.; Hickel, T.; Neugebauer, J.; Kresse, G.; Janotti, A.; Van de Walle, C. G. First-principles calculations for point defects in solids. *Rev. Mod. Phys.* **2014**, *86* (1), 253-305.

(21) Jun, A.; Yoo, S.; Gwon, O.-h.; Shin, J.; Kim, G. Thermodynamic and electrical properties of Ba0.5Sr0.5Co0.8Fe0.2O3−δ and La0.6Sr0.4Co0.2Fe0.8O3−δ for intermediate-temperature solid oxide fuel cells. *Electrochimica Acta* **2013**, *89*, 372-376.

(22) Lee, B.; Stoumpos, C. C.; Zhou, N.; Hao, F.; Malliakas, C.; Yeh, C.-Y.; Marks, T. J.; Kanatzidis, M. G.; Chang, R. P. H. Air-Stable Molecular Semiconducting Iodosalts for Solar Cell Applications: Cs2SnI6 as a Hole Conductor. *J. Am. Chem. Soc.* **2014**, *136* (43), 15379-15385.

(23) Kobayashi, T.; Wang, S.; Dokiya, M.; Tagawa, H.; Hashimoto, T. Oxygen nonstoichiometry of Ce1−ySmyO2−0.5y−x (y=0.1, 0.2). *Solid State Ion.* **1999**, *126* (3), 349-357.

(24) Chueh, W. C.; Haile, S. M. Electrochemical studies of capacitance in cerium oxide thin films and its relationship to anionic and electronic defect densities. *Phys. Chem. Chem. Phys.* **2009**, *11* (37), 8144-8148.

(25) Mizusaki, J.; Tagawa, H.; Naraya, K.; Sasamoto, T. Nonstoichiometry and thermochemical stability of the perovskite-type La1−xSrxMnO3−δ. *Solid State Ion.* **1991**, *49*, 111-118.
