# Supplementary material for: Halide Perovskites Breathe Too: The Iodide–Iodine Equilibrium and Self-Doping in Cs2SnI6
Source: ACS Cent Sci. 2024 Apr 2;10(4):907–19. doi: 10.1021/acscentsci.4c00056 (PMC11046464; doi:10.1021/acscentsci.4c00056)
Supplement: Supplementary file 2 — oc4c00056_si_002.pdf [file oc4c00056_si_002.pdf]

Vigil(2024)_supporting_code_diffusionfitting

January 11, 2024

1 Supplementary Information: Code used in the diffusion model fitting

1.1 Authors: Julian A. Vigil, Nathan R. Wolf, Adam H. Slavney

1.1.1 Halide perovskites breathe too: The iodide-iodine equilibrium and self-doping in Cs2SnI6

Julian A. Vigil,†,‡ Nathan R. Wolf,† Adam H. Slavney,† Roc Matheu,† Abraham Saldivar Valdes,† Aaron Breidenbach,§,# Young S. Lee,¶,# and Hemamala I. Karunadasa†,#,*

† Department of Chemistry, Stanford University, Stanford, California 94305, United States; ‡ Department of Chemical Engineering, Stanford University, Stanford, California 94305, United States; § Department of Physics, Stanford University, Stanford, California 94305, United States; ¶ Department of Applied Physics, Stanford University, Stanford, California 94305, United States; # Stanford Institute for Materials and Energy Sciences, SLAC National Laboratory, Menlo Park CA 94025; *e-mail: hemamala@stanford.edu

1.2 Fitting to the 1D Diffusion Model

This notebook handles the fitting of the pre-processed decay data (i.e. measured VDP resistance converted to conductivity) to the 1D vacancy diffusion model. To do this, we provide pre-processed data, constants including the crystal thickness and mobility (constant for now), and initial guesses/bounds for the three variable parameters in the model, namely the diffusion coefficient (D) and carrier concentrations at the surface (Cs) and in the bulk (Cb). At present, the code assumes no exchange happens at the back-surface of the crystal and thus the full crystal thickness is used in the model. In a circumstance where the crystal exchange problem becomes symmetric (i.e. exchange at both surfaces), then the thickness should be halved and the solution is symmetric about the midpoint.

The diffusionfit() function performs the following tasks: 1. Loads and cleans .csv data files from the output of pre-processing 2. Defines the degassing diffusion model and related residual and statistics used in concert with least_squares 3. Performs the fit and returns optimized parameters and the model fit 4. Plots the model fit and measured conductivity and exports the processed data alongside the associated parameters 5. Linearization routine to fit the logarithmic prortion of the decay

Subprograms can be run independently to examine data or loaded and run in full (diffusionfit()) to complete the fitting routine.

1.3 I. Importing packages and defining parameters

[ ]:
```
## packages:
import pandas as pd
import numpy as np
import matplotlib.pyplot as plt
import matplotlib as mpl
mpl.rcParams['font.sans-serif'] = "Arial"
mpl.rcParams['font.size'] = 14

import scipy
from scipy.optimize import least_squares
from sklearn.linear_model import LinearRegression
from scipy import stats

## parameters:
runname = 'NRW7-118' # Main run name for the collection of measurements,␣
↪associated with the crystal, notebook code
mobilityconstant = True # Is the mobility constant? True (const. mobility, RT␣
↪val) OR False (variable mobility)
runlist = np.array([9,10,12]) # Populate with the run numbers for all␣
↪off-gassing traces, i.e. GEIS9,10
runint = 3 # Tell me which trace to fit, i.e. 1 = first, 2 = second, etc.

runnum = runlist[(runint-1)] # Defines runnum with the current GEIS run number
thickness = 500e-6 # Measured crystal thickeness in meters
lb = np.array([0.5e-10, 1e13, 1e13]) # Reasonable lower bounds to use in␣
↪fitting for D, Cs, Cb
ub = np.array([1e-8, 1e16, 1e16]) # Reasonable upper bounds to use in fitting␣
↪for D, Cs, Cb
p = np.array([1.00358144e-09, 3.05245265e+15, 1.78559490e+15]) # Reasonable␣
↪initial guesses to use in fitting for D, Cs, Cb
```

1.4 II. Defining subprograms

1.4.1 0. Dealing with the mobility

[ ]:
```
## The mobilityhandle() function ...

def mobilityhandle(mobilityconstant, runint, thickness):
    if mobilityconstant == True:
        mobility = 7.17
    if mobilityconstant == False:
        mobilitycalc = pd.read_csv('%s_mobilitycalc.csv'%runname)
        mobility = mobilitycalc.iloc[(runint-1)]['mobility']
    c = np.array([thickness,mobility,50]) # Constants for fitting: thickness,␣
↪mobility, number of terms
```

```
    return c, mobility
```

1.4.2 1. Importing and cleaning the pre-processed data

[ ]:
```
## The load_data() function loads the pre-processed data from the file of␣
↪choice (GEIS run number = runnum = num)
## and returns a clean data frame with unnecessary column dropped

def load_data(num):
    predata = pd.read_csv('GEIS%s.csv'%num)
    predata = predata.drop('Unnamed: 0',1)
    return predata
```

1.5 ### 2. Degassing diffusion model and related residual, statistic definitions

This script is used to fit the change in conductivity over time of a degassing experiment. Using a least-squares algorithm it fits the data to the following equation:

$$\sigma = e|z|\mu \left[c_b + (c_s - c_b) \left(1 - \frac{8}{\pi^2} \sum_{i=0}^{\infty} \frac{1}{(2i+1)^2} \exp\left(\frac{\pi^2 t D}{L^2} (2i+1)^2 \right) \right) \right]$$

where: σ = conductivity [S/cm]; z = charge of the defect; e = electron charge [C]; μ = carrier mobility [cm2/Vs]; c_s/c_b = defect concentration at surface/bulk [cm-3]; t = time [s]; D = diffusion coefficient of defect [cm2/s]; L = thickness of the crystal [m]

This is an analytical solution to the Fickian diffusion problem and is valid under the following assumptions: 1. The mobile defect is either directly responsible for the observed conductivity or is the major compensating defect for the electrically active species.
2. The defect reaction at the surface is not rate-limiting and there is a fixed concentration of defects at the surface.
3. At t= 0 the crystal has a uniform defect concentration. 4. No defect transport occurs at the back crystal surface.

Equation taken from Maier, J. Ch 4. Kinetics and Irreversible Thermodynamics. In _Physical Chemistry of Ionic Materials; John Wiley & Sons; (2004). *p* 312-319.

[ ]:
```
## The degass_model() function calculates the change in conductivity expected␣
↪for a given set of parameters
## for a degassing experiment under the above conditions. Inputs: p =␣
↪parameters; c = constants; t = time
## p[0] = D = Diffusion Coefficient [cm^2/s]; p[1] = C_s = Carrier␣
↪concentration at the surface [cm^-3]
## p[2] = C_b = Carrier concentration in the bulk [cm^-3]; c[0] = L = Crystal␣
↪thickness [m];
```

```
## c[1] = mu = Carrier mobility [cm^2/Vs]; c[2] = n_t = number of terms to␣
↪calculate

def degass_model(p, c, t):
    el = 1.602e-19                    # electron charge in [C]
    Lnew = c[0]*100                   # Converts L to [cm]
    tau = (Lnew**2)/(np.pi**2*p[0]) # Calculates time constant of the system
    sum1 = np.zeros((t.size))
    for i in range(int(c[2])):
        sum1 = sum1 + 1/((2*i+1)**2)*np.exp(-t/tau*(2*i+1)**2)
    cond = el*c[1]*(p[2]+(p[1]-p[2])*(1-8/(np.pi**2)*sum1))
    return cond

## The residual() function calculates the residual (difference) between the␣
↪measured conductivity (y) and
## the calculated conductivity [degass_model(p,c,t)], which is used during␣
↪fitting to minimize the SSR

def residual(p, c, t, y):
    return (degass_model(p,c,t) - y)

## the fit_stats() function calculates statistics for the fit to the degassing␣
↪model. Inputs: results = output
## of the least_squares procedure; c = constants; x = time (or generally, indep.
↪ var.); y = measured conductivity

def fit_stats(results, c, x, y):
    # Calculating the adjusted R-squared statistic
    tSum_sq = np.var(y) # Computing total sum of squares (variance) of y-data
    rSum_sq = (residual(results.x,c,x,y)**2).sum()/(len(x)-3) # Computing␣
↪residual sum of squares
    adjR_sq = 1 - rSum_sq/tSum_sq
    # Calculating the covariance matrix from the fit Jacobian
    covar = np.matrix.getI(np.matrix.transpose(results.jac)*np.matrix(results.
↪jac))
    covar = covar*rSum_sq
    # Calculating the parameter std. deviations from the diagonal of the␣
↪covariance matrix
    stdError = np.array(np.diagonal(covar))**0.5
    # Calculating the correlation matrix from the covariance matrix and␣
↪standard errors
    corr = np.diagflat(1/stdError)*covar*np.diagflat(1/stdError)
    return print('Adjusted R2 = ', adjR_sq,
                 '\n Standard Deviations of D, Cs, & Cb:\n', stdError,
                 '\n Correlation matrix of parameters:\n', corr)
```

1.5.1 3. Main fitting function and data-fit comparison

[ ]:
```
## The main_fitter() function performs the fit by optimizing the parameters D,␣
↪Cs, Cb (within p) given the constants
## in c and returns the optimized values for D, Cs, Cb within resid.x

def main_fitter(c, p, data, lb, ub):
    resid = least_squares(residual, p, args=(c,data.iloc[:,0],data.iloc[:,1]),
                          bounds=(lb,ub), xtol=1e-15, x_scale="jac",␣
↪verbose=1)
    return resid

## The build_comparison() function simply adds a column to the existing␣
↪dataframe, now re-named 'postdata', that
## calculates the model fit given optimized parameters stored within resid.x

def build_comparison(data, resid, c):
    postdata = data
    postdata['fitconductivity/s/cm'] = degass_model(resid.x,c,postdata['time/
↪s'])
    return postdata
```

1.5.2 4. Comparison plot generator and output for the fit values and data-fit comparison

[ ]:
```
## The plot_cond() function plots the conductivity calculated from the measured␣
↪VDP resistance (blue scatter) vs. the
## model fit (red line). x-axis is time in s.

def plot_cond(postdata):
    fig = plt.figure(figsize=(8,5))
    plt.plot(postdata['time/s'],postdata['conductivity/s/
↪cm'],'bo',markerfacecolor='white')
    plt.plot(postdata['time/s'],postdata['fitconductivity/s/
↪cm'],'r',linewidth=3)
    plt.xlabel('time / s'); plt.ylabel('conductivity/s/cm');
    plt.tight_layout()
    #plt.savefig('fit_varmob_GEIS18', dpi=300)

## The output_data() function saves the postdata frame with the added model fit␣
↪(_model) and the optimized
## parameters (_fitvals) to the directory that the notebook is in. It is named␣
↪as the GEIS file that was input.

def output_data(postdata, resid, num, L):
    postdata.to_csv('GEIS%s'%num + '_model.csv')
```

```
    fitvals = pd.DataFrame(columns=('D/cm2/s','Cs/cm-3','Cb/cm-3'))
    fitvals.loc[0] = resid.x
    fitvals['tau/s'] = (L*100)**2/np.pi**2/fitvals.iloc[0]['D/cm2/s']
    fitvals.to_csv('GEIS%s'%num + '_fitvals.csv')
    return fitvals
```

1.6 ### 5. Linearize to the logarithmic portion and plot

Let us now consider the expected behavior of the conductivity vs. time. Based on the functional form of the model as defined above,

$$\sigma = e|z|\mu \left[c_b + (c_s - c_b)\left(1 - \frac{8}{\pi^2}\sum_{i=0}^{\infty}\frac{1}{(2i+1)^2}\exp\left(\frac{\pi^2 tD}{L^2}(2i+1)^2\right)\right)\right]$$

at short times [i.e. t « τ, where $\tau = L^2/(\pi^2 D)$], the function follows a $t^{1/2}$ dependence:

$$\frac{\sigma(\mathrm{t}) - \sigma_b}{\sigma_s - \sigma_b} \simeq \frac{4}{\pi^{3/2}}\sqrt{\frac{\mathrm{t}}{\tau}}$$

however, at long times (i.e. t ~ τ), the function follows a logarithmic dependence:

$$M_\sigma \equiv \frac{\sigma(\mathrm{t}) - \sigma_s}{\sigma_b - \sigma_s} = \frac{8}{\pi^2}\exp\left(\frac{-\mathrm{t}}{\tau}\right)$$

where: σ = conductivity [S/cm]; z = charge of the defect; e = electron charge [C]; μ = carrier mobility [cm2/Vs]; c_s/c_b = defect concentration at surface/bulk [cm-3]; σ_s/σ_b = conductivity at surface/bulk [S/cm]; t = time [s]; τ = time constant [s]; D = diffusion coefficient of defect [cm2/s]; L = thickness of the crystal [m]

Equation taken from Maier, J. Ch 4. Kinetics and Irreversible Thermodynamics. In _Physical Chemistry of Ionic Materials; John Wiley & Sons; (2004). *p* 312-319.

[ ]:
```
## the linearize() function linearizes the conductivity data calculate from␣
↪measured VDP resistance, as described
## above, and fits a line to calculate the diffusion coefficient from the␣
↪"linear portion" of the data

def linearize(data, fitvals, mu):
    # Calculating the quotient M and logM
    linfit = data.drop(['Temperature','fitconductivity/s/cm'],1)
    linfit['quotient'] = (linfit['conductivity/s/cm']/(1.602e-19*mu)-fitvals.
↪iloc[0]['Cs/cm-3'])/(fitvals.iloc[0]['Cb/cm-3']-fitvals.iloc[0]['Cs/cm-3'])
    linfit = linfit[linfit > 0].dropna()
    linfit['shiftlogM'] = np.log(linfit['quotient'])-np.log(8/np.pi**2)
```

```
    # Performing the linear regression
    model = LinearRegression(fit_intercept=False).fit(linfit['time/s'].values.
↪reshape(-1,1),linfit['shiftlogM'].values.reshape(-1,1))
    linfit['linfit'] = model.coef_.item()*linfit['time/s']

    # Plotting, option to save fig, csv of the linearized data and fit, and␣
↪adding D from linearization (Dlin) to table
    fig = plt.figure(figsize=(8,5))
    plt.plot(linfit['time/s'],linfit['shiftlogM'],'bo',markerfacecolor='white')
    plt.plot(linfit['time/s'],linfit['linfit'],'r',linewidth=3)
    plt.xlabel('time / s'); plt.ylabel('log(M)');
    plt.tight_layout()
    #plt.savefig('lin_varmob_GEIS18', dpi=300)
    #linfit.to_csv('GEIS%s'%runnum + "_linfit.csv")
    fitvals['Dlin/cm2/s'] = -model.coef_.item()*(thickness*100/np.pi)**2
    fitvals.to_csv('GEIS%s'%runnum + '_fitvals.csv')
    return linfit
```

1.7 III. Main function for diffusion fitting

[ ]:
```
## diffusionfit() is the main function of the notebook that will run all (or␣
↪some, at discretion) of the above
## subprograms.

def diffusionfit(runnum, thickness, lb, ub, p):
    c, mobility = mobilityhandle(mobilityconstant, runint, thickness)
    predata = load_data(runnum)
    resid = main_fitter(c, p, predata, lb, ub)
    print(resid.x)
    fit_stats(resid,c,predata['time/s'],predata['conductivity/s/cm'])
    postdata = build_comparison(predata, resid, c)
    plot_cond(postdata)
    fitvals = output_data(predata, resid, runnum, thickness)
    linfit = linearize(predata, fitvals, mobility)
```

[ ]:
```
## do the fitting

diffusionfit(runnum, thickness, lb, ub, p)
```
